# Transcription Factors STAT3 and MYC Are Key Players of Human Platelet Lysate-Induced Cell Proliferation

**DOI:** 10.3390/ijms232415782

**Published:** 2022-12-13

**Authors:** Michaela Oeller, Heidi Jaksch-Bogensperger, Markus Templin, Renate Gehwolf, Eva Rohde, Katharina Schallmoser, Sandra Laner-Plamberger

**Affiliations:** 1 Department of Transfusion Medicine, Salzburger Landeskliniken (SALK) & Paracelsus Medical University Salzburg (PMU), 5020 Salzburg, Austria; 2 Spinal Cord Injury and Tissue Regeneration Center Salzburg, PMU Salzburg, 5020 Salzburg, Austria; 3 Department of Obstetrics and Gynaecology, Salzburger Landeskliniken (SALK) & Paracelsus Medical University Salzburg (PMU), 5020 Salzburg, Austria; 4 NMI Natural and Medical Sciences Institute at the University of Tuebingen, 72770 Reutlingen, Germany; 5 Institute of Tendon and Bone Regeneration, PMU Salzburg, 5020 Salzburg, Austria; 6 Austrian Cluster for Tissue Regeneration, 1200 Vienna, Austria; 7 GMP Unit, Spinal Cord Injury and Tissue Regeneration Center Salzburg, PMU Salzburg, 5020 Salzburg, Austria

**Keywords:** human platelet lysate (HPL), signal transducer and activator of transcription 3 (STAT3), MYC, human mesenchymal stromal cells (MSCs), proliferation

## Abstract

Human platelet lysate (HPL) is an efficient alternative for animal serum supplements, significantly enhancing stromal cell proliferation. However, the molecular mechanism behind this growth-promoting effect remains elusive. The aim of this study was to investigate the effect of HPL on cell cycle gene expression in different human stromal cells and to identify the main key players that mediate HPL’s growth-enhancing effect. RT-qPCR and an antibody array revealed significant upregulation of cell cycle genes in stromal cells cultured in HPL. As HPL is rich in growth factors that are ligands of tyrosine kinase receptor (TKR) pathways, we used TKR inhibitors and could significantly reduce cell proliferation. Genome profiling, RT-qPCR and Western blotting revealed an enhanced expression of the transcription factors signal transducer and activator of transcription 3 (STAT3) and MYC, both known TKR downstream effectors and stimulators of cell proliferation, in response to HPL. In addition, specifically blocking STAT3 resulted in reduced cell proliferation and expression of cell cycle genes. Our data indicate that HPL-enhanced cell proliferation can, at least in part, be explained by the TKR-enhanced expression of STAT3 and MYC, which in turn induce the expression of genes being involved in the promotion and control of the cell cycle.

## 1. Introduction

The therapeutic potential of so-called human ‘mesenchymal’ stromal cells (‘MSCs’) [1,2] is currently being tested in numerous clinical trials worldwide, mainly targeting muscle, bone and cartilage defects, neurological and cardiovascular disorders, wound and injury treatment and recently also coronavirus disease 2019 (COVID-19) [3]. As sufficient cell numbers are required for therapeutic interventions, the ex vivo propagation of human stromal cells from different tissue sources is an important prerequisite. Usually fetal bovine serum (FBS) is used to support cell proliferation [4,5]. However, this bears the risks of transmitting bovine pathogens including prions, and may induce undesirable immunologic reactions [4,6,7,8]. Therefore, the European Medicines Agency (EMA) has discouraged the use of FBS, recommending the use of non-animal serum supplements [9,10,11]. 

Chemically defined media may circumvent the need for serum supplementation. However, to date, cost-effective chemically defined media optimally supporting cell growth are still not available [8,12]. A valid alternative for animal sera is human platelet lysate (HPL) [13,14,15]. HPL can be produced from outdated platelet concentrates manufactured at blood banks worldwide to treat thrombocytopenic patients. The lysis of platelets is usually induced by either repeated freeze/thaw cycles, sonication or treatment with detergents [16]. This results in the release of numerous growth factors and cytokines, which are stored in the platelets’ granules, into the plasma or additive solution. After quality control tests including the screening for endotoxin and mycoplasma, HPL is used as efficient medium supplement for good manufacturing practice (GMP)-compliant 2D cell culture [16] as well as hydrogel-based 3D cell cultivation [17]. HPL has been applied for the cultivation of a variety of cell types including primary cells (e.g., osteoblasts, chondrocytes, stromal cells and endothelial cells) and established cell lines (e.g., HaCaT, JURKAT, HeLa and MCF-7) [12]. However, HPL was not only shown to support cell growth, but also to significantly enhance cell proliferation when compared to FBS [5,18]. In our previous study [19], various modified HPL products supported cell proliferation more efficiently than FBS. This effect may be explained by alpha granule-derived growth factors and cytokines such as platelet-derived growth factor (PDGF), fibroblast growth factor (FGF) or epidermal growth factor (EGF) [20,21], detected in comparable concentrations independently of HPL production mode [19]. Recently, a flow cytometry-based cell cycle analysis of human umbilical cord-derived stromal cells cultured in different concentrations of HPL revealed a progressive decrease in the G0/G1 phase percentage and an increase in the S and G2/M phases, indicating dose-dependently enhanced cell cycle progression [22].

As the downstream molecular mechanisms leading to enhanced cell proliferation are still elusive, the aim of this study was (i) to investigate the effect of HPL on cell cycle target gene expression and (ii) to identify the main molecular key players mediating the HPL-growth promoting effects. Stromal cells derived from human bone marrow (BM), umbilical cord (UC) and white adipose tissue (WAT) were analyzed. Using RT-qPCR and a cell cycle-specific antibody array, we showed that HPL significantly affected cell cycle-specific protein expression when compared to FBS-based culture. We further found that blocking specific tyrosine kinase receptor (TKR) pathways lead to a significant reduction of HPL-induced cell proliferation. Additionally, the TKR downstream effector signal transducer and activator of transcription 3 (STAT3) and the STAT3 downstream target MYC, both known to support cell proliferation, were significantly upregulated in HPL-cultured stromal cells. Furthermore, we observed a significantly reduced cell proliferation and cell cycle target gene expression when specifically inhibiting STAT3 in HPL-based culture. Our data indicate that HPL-induced cell proliferation can, at least in part, be explained by enhanced TKR signaling and thus elevated expression of the TKR downstream effectors STAT3 and MYC, which in turn induce the expression of cell cycle promoting target genes such as *cyclin A1* (*CCNA1*), *cyclin D1* (*CCND1*) and *cyclin-dependent kinase 2* (*CDK2*).

## 2. Results

### 2.1. HPL Induces the Expression of Target Genes Promoting Cell Cycle Progression and of Genes Involved in Repair and Cell Cycle Arrest

**Figure 1:**
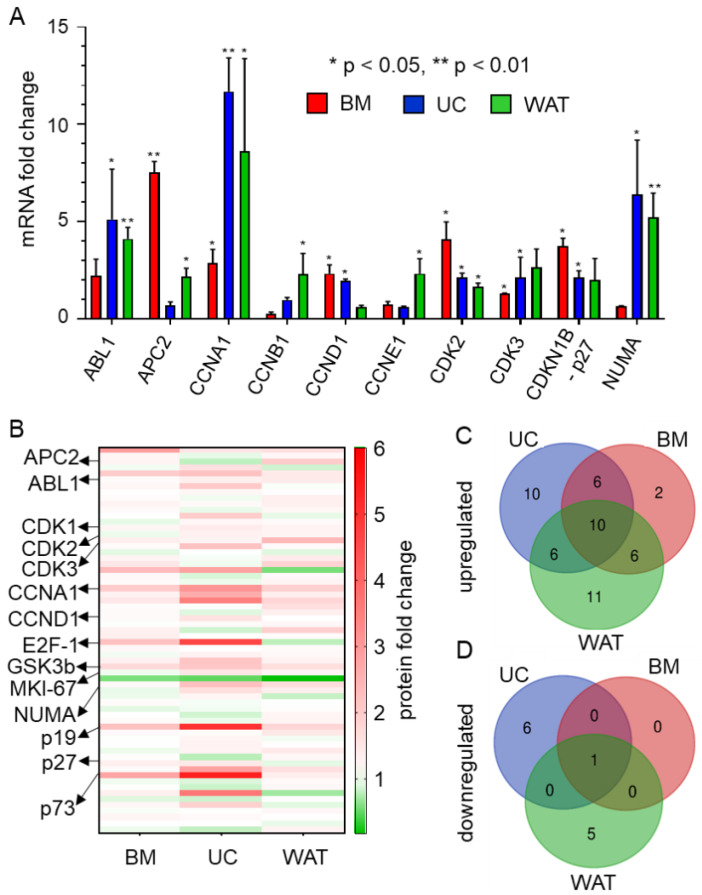
HPL-based culture conditions induce the expression of cell cycle-specific target genes. (**A**) RT-qPCR reveals that mRNA expression is significantly upregulated when BM- (red), UC- (blue) and WAT-derived stromal cells (green) are cultured in HPL. Data are shown as mean fold change values ± SD of biological triplicates measured in technical duplicates. *ABL proto-oncogene 1 (ABL1), adenomatous polyposis coli protein 2 (APC2), cyclin A1 (CCNA1), cyclin B1 (CCNB1), cyclin D1 (CCND1), cyclin E1 (CCNE1), cyclin-dependent kinase 2 and 3 (CDK2, 3), cyclin-dependent kinase inhibitor 1B (CDKN1B, p27) and nuclear mitotic apparatus protein 1 (NUMA)*. (**B**) Heat map of cell cycle-specific antibody array depicting the up- (shown in red) and downregulated (shown in green) protein expression levels of 64 different specific cell cycle targets of stromal cells cultured in HPL compared to FBS. Data depicted are mean fold change values (HPL versus FBS) of biological triplicates measured in quadruplicates. (**C**,**D**) Venn diagrams of significantly up- and downregulated cell cycle-associated proteins show distinct source dependence, but also common protein expression patterns for BM-, UC- and WAT-derived stromal cells cultured in HPL.

In order to identify whether the expression of cell cycle target genes is influenced by HPL culture in BM-, UC- and WAT-derived stromal cells, RT-qPCR was carried out. As shown in Figure 1A, the mRNA expression of *CCNA1* and *CDK2* was significantly enhanced in all stromal cell types, while other cell cycle-associated genes revealed cell source-dependent upregulation in HPL- compared to FBS-based culture. Our findings were corroborated on the protein level by a specific cell cycle antibody microarray (Figure 1B, Table S1). This array furthermore revealed that more genes were upregulated than downregulated. Twenty-four proteins were upregulated in BM-, 32 in UC- and 33 in WAT-derived stromal cells, while one protein was downregulated in BM-, 7 in UC- and 6 proteins in WAT-derived stromal cells. Ten protein targets were upregulated in all three stromal cell sources. In contrast, all stromal cells revealed the decreased expression of only one protein, namely mitochondria ab-2 (Figure 1C,D, Table S2).

CCNA1 and cyclin-dependent kinase 1 (CDK1) protein expression was significantly upregulated in all types of stromal cells (Figure 1B and Tables S2 and S3). Additionally CDK2, which also interacts with CCNA1 during cell cycle progression, was significantly upregulated in UC- and WAT-derived stromal cells. BM- and UC-derived stromal cells revealed an elevated protein expression of transcription factor E2F-1, which regulates cell cycle progression through the G1/S transition, but is also important for DNA repair and apoptosis. The protein expression of other cyclins such as cyclin E1 (CCNE1), cyclin B1 (CCNB1) and cyclin D1 (CCND1) and cyclin-dependent kinase 3 and 4 (CDKs 3 and 4) was upregulated in a tissue source dependent manner. UC- and WAT-derived stromal cells also showed upregulated expression of the proteins involved in the formation and organization of the mitotic spindle during cell division such as nuclear mitotic apparatus protein 1 (NUMA) and marker of proliferation Ki-67 (MKI-67). In addition to proteins enhancing cell cycle progression, proteins were also involved in repair mechanisms and cell cycle arrest such as glycogen synthase kinase 3 beta (GSK3B); p73 and p19 were upregulated in HPL-cultured stromal cells.

### 2.2. Specific Inhibitors of Tyrosine Kinase Receptor Pathways Reduce HPL-Induced Proliferation

**Figure 2:**
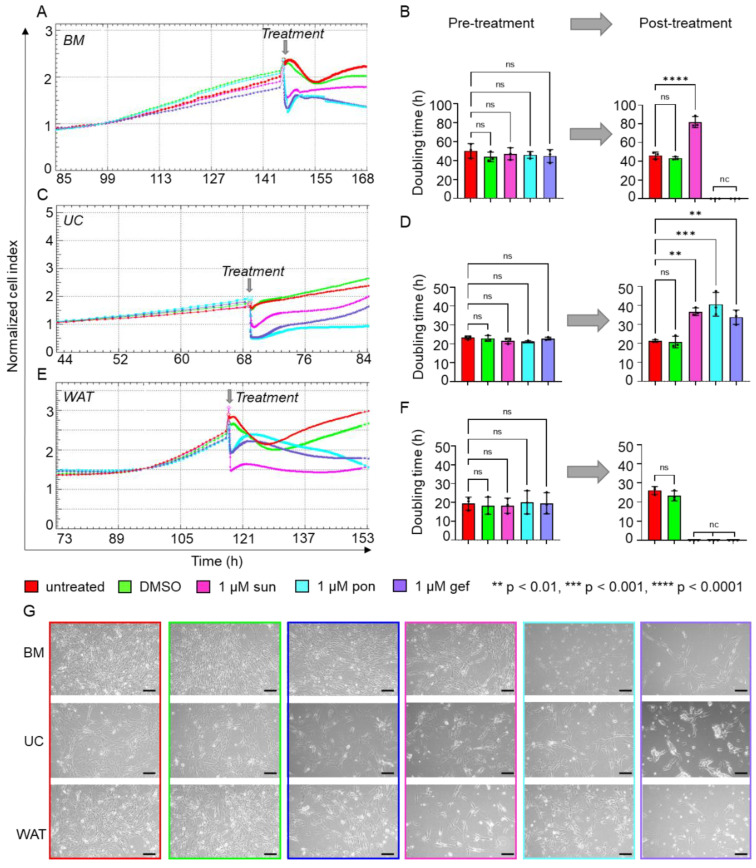
Inhibitors of TKR signaling significantly reduce HPL-induced proliferation. xCELLigence impedance measurements of (**A**) BM-, (**C**) UC- and (**E**) WAT-derived stromal cells before and after TKR inhibitor treatment. After reaching a normalized cell index of at least 2, treatment with either 1 µM sunitinib (sun, magenta), 1 µM ponatinib (pon, cyan) or 1 µM gefitinib (gef, violet) was carried out (treatment time indicated as grey arrow). Untreated cells (red) or stromal cells treated with DMSO only (green) served as control. Our data reveal significantly reduced or completely abolished cell proliferation after TKR inhibitor treatment, as indicated. xCELLigence impedance measurements are depicted as mean normalized cell index over time of one representative donor for each tissue source measured in technical quadruplicates. (**B**,**D**,**F**) The doubling time in hours (h) was calculated for each tissue source before and 24 h after the treatment. Data shown are mean values ± SD of three biological replicates measured in technical quadruplicates; nc = not calculable, ns = not significant. (**G**) For each cell type, cell morphology was documented 8 h after inhibitor treatment as indicated. One representative is shown for each cell type. Scale bar = 200 µm.

HPL is known to contain various mitogenic growth factors, such as PDGF, FGF and EGF, but also vascular endothelial growth factor (VEGF) and transforming growth factor (TGF) [13,21], which bind to their specific tyrosine kinase receptors and thus activate these signaling pathways. As a next step, we used the specific TKR cascade inhibitors gefitinib, ponatinib and sunitinib. Prior to TKR-inhibitor treatment, stromal cells revealed similar normalized cell indices (Figure 2A,C,E) and doubling times (Figure 2B,D,F) depending on their tissue origin as monitored by xCELLigence impedance measurements. Blocking VEGF/PDGF signaling by sunitinib, we observed reduced normalized cell indices and significantly increased doubling times for BM- (Figure 2A,B) and UC-derived stromal cells (Figure 2C,D) when compared to untreated cells or cells treated with DMSO only. The proliferation of WAT-derived stromal cells was completely abolished by 1 µM sunitinib as shown by the constant decrease in normalized cell index (Figure 2E), indicating that this treatment leads to increased cell death (Figure 2F,G). The inhibition of TKR signaling by ponatinib and gefitinib abolished proliferation for BM- (Figure 2A,B,G) and WAT-derived stromal cells (Figure 2E,F,G). Furthermore, we observed significantly lower proliferation rates for UC-derived stromal cells (Figure 2C,D,G) as indicated by significantly prolonged doubling times.

### 2.3. Transcription Factors STAT3 and MYC Are Upregulated in Stromal Cells in Response to HPL-Culture

**Figure 3:**
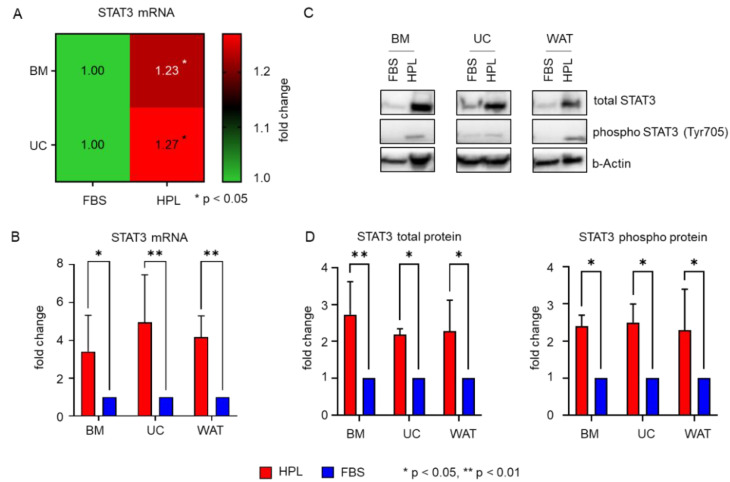
HPL mediates significant activation of STAT3 expression. (**A**) Heat map of genome expression profiling showing significantly enhanced expression levels of STAT3 in BM- and UC-derived stromal cells when cultured in HPL compared to FBS. Data shown are mean fold change expression values ± SD of three biological replicates measured in technical duplicates. (**B**) RT-qPCR reveals significantly elevated mRNA expression for all cell types when cultured in HPL (red bar) compared to FBS (blue bar). Data shown are mean fold change values ± SD of three biological replicates measured in duplicates. (**C**) Western blot demonstrates that STAT3 total protein and phospho-STAT3 (Tyr705) are elevated in HPL compared to FBS-culture. One representative for each cell type is shown. (**D**) Quantitative analysis of Western blots (HPL: red bars, FBS: blue bars). Data shown are mean fold change values ± SD of three biological replicates normalized to b(eta)-Actin expression.

We next asked whether there is a common downstream effector of TKR pathways that might confer the intracellular HPL-mediated growth stimulus. Genome expression profiling of HPL and FBS-cultured BM- and UC-derived stromal cells revealed that the transcription factor *STAT3*, a downstream effector of different TKR signaling cascades [23,24,25,26], was significantly elevated in HPL-cultured stromal cells (Figure 3A). This finding was corroborated by RT-qPCR (Figure 3B), also revealing significantly elevated *STAT3* mRNA levels in HPL-cultured WAT-derived stromal cells. Quantitative Western blots confirmed this finding on the protein level for total STAT3, but also for phospho-STAT3 (Tyr705) (Figure 3C,D).

**Figure 4:**
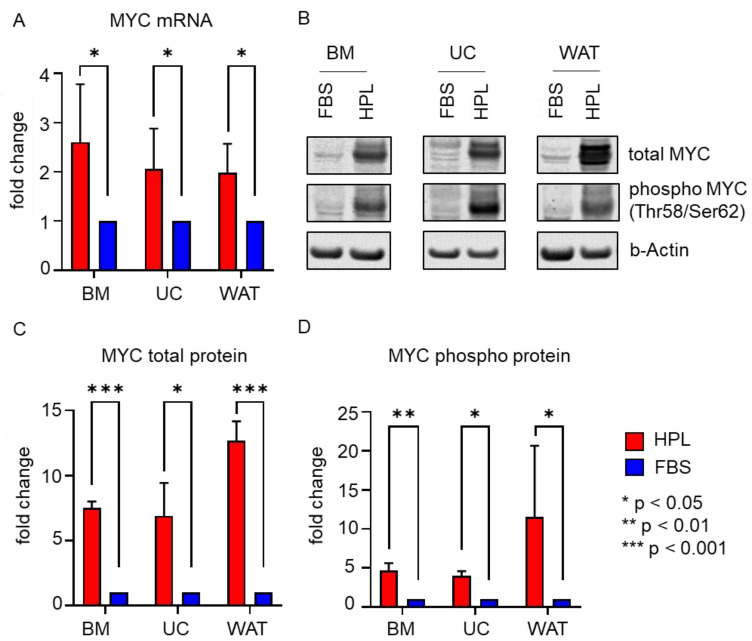
MYC mRNA and protein expression are significantly enhanced in HPL-cultured stromal cells. (**A**) RT-qPCR shows significantly enhanced *MYC* mRNA expression in HPL-culture (red bar) when compared to FBS (blue bar). Data shown are mean fold change values ± SD of three biological replicates measured in duplicates. (**B**) Western blot revealed significantly higher MYC total protein and phosphoprotein (Thr58/Ser62) expression in HPL-cultured stromal cells. One representative for each cell type is shown. (**C**,**D**) Quantitative analysis of Western blots depicted as mean fold change values ± SD of three biological replicates normalized to b(eta)-Actin expression (HPL: red bars, FBS: blue bars).

Furthermore, we found that the expression of *MYC*, a direct transcriptional target gene of *STAT3* [27,28], was enhanced in the presence of HPL. As depicted in Figure 4A, all three stromal cell types revealed significantly elevated *MYC* mRNA levels when cultured in HPL compared to FBS. Additionally, MYC total protein and MYC phospho protein (Thr58/Ser62) expression were significantly enhanced in HPL-culture, as demonstrated by quantitative Western blots (Figure 4B–D).

### 2.4. Blocking STAT3 Dimerization and Inhibition of STAT3 Phosphorylation Significantly Decrease HPL-Induced Cell Proliferation

**Figure 5:**
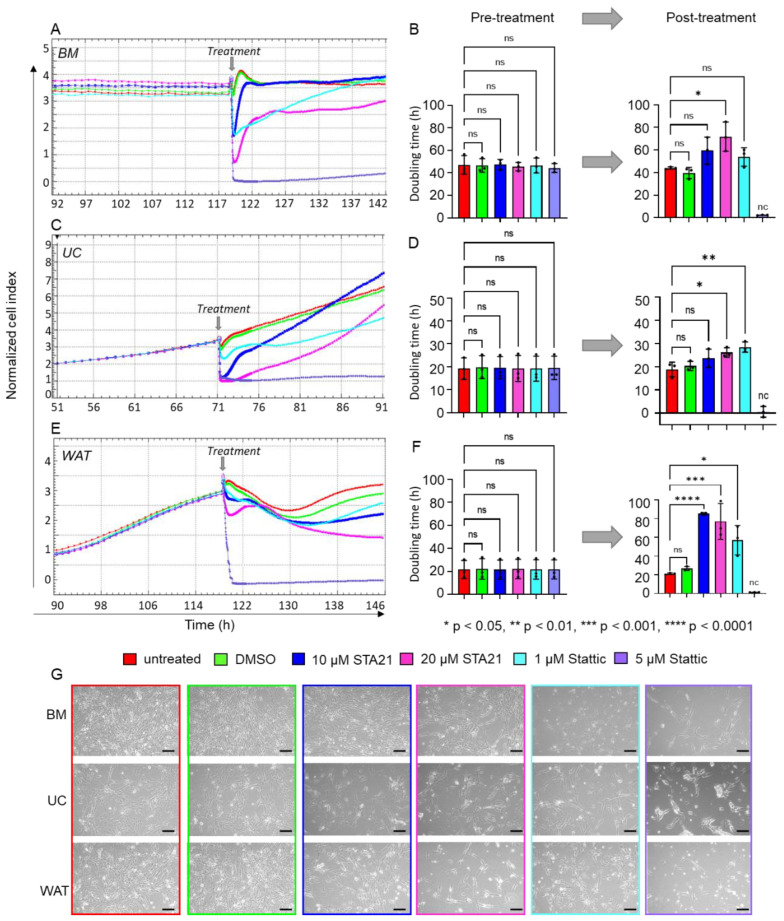
Specific inhibition of STAT3 significantly decreased HPL-mediated proliferation of stromal cells. xCELLigence impedance measurements of (**A**) BM-, (**C**) UC- and (**E**) WAT-derived stromal cells. Treatment with the specific STAT3 inhibitors STA21 and Stattic was carried out at two different concentrations (treatment times indicated as grey arrow). Our data revealed substantially reduced cell proliferation after the treatment with both lower (10 µM STA21 in blue, 1 µM Stattic in cyan) and higher (20 µM STA21 in magenta, 5 µM Stattic in violet) concentrations. Untreated (red) cells or stromal cells treated with DMSO only (green) served as control. Data shown are xCELLigence impedance measurements depicted as mean normalized cell index over time of one representative cell donor for each source measured in technical quadruplicates. (**B**,**D**,**F**) The doubling time in hours (h) was calculated for each stromal cell type before and 24 h after the treatment. Data shown are mean doubling time values ± SD, given in hours, of three biological replicates measured in technical quadruplicates; nc = not calculable, ns = not significant. (**G**) Cell morphology was documented 8 h after the treatment with the inhibitor and respective concentration as indicated. One representative is shown for each stromal cell type. Scale bar = 200 µm.

As STAT3 expression and activity seem to be elevated in HPL-based cell culture, we used two different STAT3 specific inhibitors to investigate its role for the growth-promoting stimulus of HPL: STA21 (10 or 20 µM) and Stattic (1 or 5 µM). Impedance measurements showed similar, normalized cell indices and doubling times prior to inhibitor treatment (Figure 5A–F). We found that the normalized cell indices of all stromal cells substantially decreased for both inhibitors and both concentrations applied (Figure 5A,C,E). Quantitative analysis revealed a significant increase in doubling times 24 h after treatment with STA21 or Stattic, indicating that cell proliferation was significantly reduced. In the presence of STA21, all stromal cells were viable and continued to proliferate. The same was observed for the treatment with 1 µM Stattic, but application of 5 µM Stattic abolished cell proliferation immediately, resulting in increasing cell death (Figure 5B,D,F,G).

**Figure 6:**
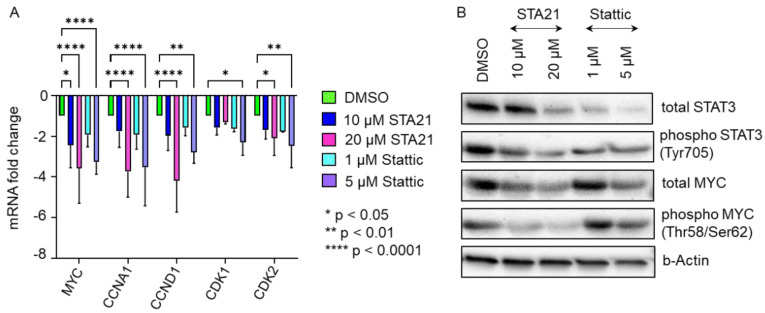
Cell cycle gene expression was significantly reduced in the presence of STAT3-specific inhibitors STA21 and Stattic. (**A**) mRNA expression levels of *MYC, CCNA1, CCND1, CDK1* and *CDK2*, which are pivotal for cell cycle progression and cell proliferation, were reduced after treatment with STAT3 specific inhibitors STA21- (blue, magenta) or Stattic- (cyan, violet) in BM-derived stromal cells. Treatment with the compound as indicated was carried out for 8 h and mRNA expression levels were compared to DMSO-treated BM-derived stromal cells (green). Data shown are mean mRNA fold change values of three biological replicates measured in technical duplicate. (**B**) Western blot analysis demonstrates substantially reduced protein expression of STAT3 and MYC in HPL-cultivated BM-derived stromal cells after STAT3-inhibitor treatment.

As our data indicated significantly decreased cell proliferation in the presence of HPL when treated with STAT3-specific inhibitors, we next asked whether the expression of STAT3/MYC target genes known to be involved in cell cycle regulation and proliferation is reduced as well. After treating HPL-cultured BM-derived stromal cells with either 10 µM STA21 or 1 µM Stattic, we found a trend towards decreased mRNA expression levels for *MYC, CCNA1, CCND1, CDK1* and *CDK2*. Increasing the concentrations to 20 µM STA21 or 5 µM Stattic, led to significantly decreased mRNA expression of *MYC, CCNA1*, *CCND1, CDK1* and *CDK2* (Figure 6A). Western blot analysis revealed reduced levels of total STAT3 following treatment with 20 µM STA21, 1 and 5 µM Stattic. We also found reduced protein levels of phospho STAT3 after inhibitor treatment (Figure 6B). Furthermore, we observed a decreased expression of total and phospho MYC when BM-derived stromal cells were treated with STA21. MYC expression was diminished after treatment with 5 µM Stattic, while hardly a reduction was observed after treatment with 1 µM Stattic (Figure 6B).

### 2.5. Increasing Levels of HPL Allow Restoration of STA21- and Stattic-Blocked Cell Proliferation

**Figure 7:**
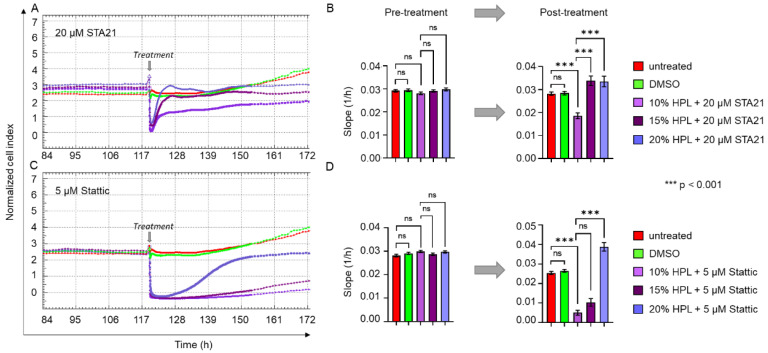
STAT3-inhibitor-induced reduction of cell proliferation can be reverted by increasing HPL concentrations. xCELLigence impedance measurements of BM-derived stromal cells grown in standard 10% HPL-supplemented medium and treated with either (**A**) 20 µM STA21 or (**C**) 5 µM Stattic. Treatment was carried out at the time indicated (grey arrow) and untreated cells (red) and DMSO only treated stromal cells (green) served as control. For the treatment with each of the two STAT3 inhibitors, three different concentrations of HPL were used: 10% (lilac), 15% (dark lilac) and 20% (violet) HPL-supplemented growth medium. Data shown are xCELLigence impedance measurements depicted as mean normalized cell index over time of one representative cell donor measured in technical quadruplicates. (**B**,**D**) The rate of change of the cell index over time (slope 1/h) ± SD was calculated before and 24 h after the treatment with 20 µM STA21 (**B**) and 5 µM Stattic (**D**); ns = not significant.

Next, we questioned whether HPL-induced cell proliferation, which is decreased by STAT3-specific inhibitors, might be restored by increasing concentrations of HPL. As indicated in Figure 7A, the decreasing effect of STA21 on the proliferative behavior of BM-derived stromal cells could indeed be reverted in the presence of 15 and 20% compared to 10% HPL. Quantitative analysis revealed a significantly reduced change of cell index over time (slope) for STA21 in 10% HPL compared to controls (untreated and stromal cells treated with DMSO). However, the normalized cell index was significantly increased in 15 and 20% HPL culture, indicating restored cell proliferation comparable to untreated/DMSO-treated stromal cells (Figure 7B). A similar observation was made for the treatment with 5 µM Stattic, with nearly restored cell proliferation rates for 20% HPL culture (Figure 7C,D).

## 3. Discussion

As a valuable alternative to FBS, HPL is increasingly used for the clinical propagation of stromal cell-based medicinal applications [29] and efforts are ongoing to define standards for HPL products [15,16]. HPL is known to not only promote human cell proliferation, but also enhance cell growth in vitro. However, the intracellular mechanisms behind this growth promoting effect are still not completely understood.

In this study, we investigated the effect of HPL-based stromal cell culture on the expression of cell cycle target genes. We found elevated expression of cyclins, their kinases and proteins being involved in the formation and organization of the mitotic spindle. Particularly the expression of CCNA1 and CDK1/2 was found to be enhanced in all three different tissue-derived stromal cell types. While CDK1 is known to be pivotal for the regulation of M-phase, CDK2 is important for regulating S/G2-phase transition when interacting with CCNA1 [30]. Similar findings were described by Yan et al., in 2021, who found elevated levels of different cytokines and CDKs including CCNA and CDK2 in HPL-cultured UC-derived stromal cells [22]. Using flow cytometry, they furthermore showed that the number of cells in the S/G2/M phase increased under HPL conditions, while the G0/G1 phase decreased. Another study from Sondergaard et al., in 2017, showed elevated levels of cyclins and decreased cell numbers in the G0/G1 fraction for WAT-derived stromal cells cultured in HPL [31]. 

Interestingly, we also observed elevated levels of genes being involved in cell cycle arrest and DNA repair such as GSK3B, p19, p73 and p27. GSK3B is a multifunctional protein kinase that may cause cell cycle arrest by suppressing the expression of critical cell cycle regulators such as CCND1. Furthermore, GSK3B is required for a functional mitotic checkpoint, which ensures proper chromosome segregation [32]. The expression of p19 leads to an inactivation of CDK4 and 6, thus preventing progression of the G1 phase [33]. p73, which also induces G1 cell cycle arrest, may also act as a repressor of G2/M regulators [34]. The role of p27 in the cell cycle depends on its phosphorylation status: On the one hand side, it opposes cell cycle progression by inhibiting the interaction between CCNE and CDKs. On the other hand, it was also shown to stabilize the interaction between CCND1 and CDK4 and to facilitate the nuclear import of this complex, thus supporting cell proliferation [35]. However, our data do not allow conclusions to be drawn on the phosphorylation status of p27. 

Our results point to an HPL-induced accelerated progression of cell cycle due to significantly elevated levels of cell cycle-promoting target genes. In addition, genes also being involved in cell cycle control, cell cycle arrest and DNA repair show enhanced expression levels in HPL-based stromal cell culture. This indicates that the HPL-induced support of cell cycle progression happens in a well-orchestrated and tightly controlled manner.

As shown previously, HPL is rich in a plethora of growth factors, including ligands for a number of TKR pathways such as PDGFR, VEGFR, FGFR and EGFR [19,21,36,37]. Thus, we used specific inhibitors of the PDGFR, VEGFR, FGFR and EGFR pathways (sunitinib, ponatinib and gefitinib) and could show that stromal cell proliferation was significantly reduced when TKR pathways were blocked. Other studies showed that activated EGFR signaling in stromal cells leads to increased proliferation, which could be reverted by gefitinib [38,39]. Furthermore, BM-derived stromal cell proliferation could significantly be decreased by blocking VEGFR/PDGFR signaling by sunitinib [40]. In 2012, Fekete at al. revealed that specific neutralizing antibodies against PDGF-BB, bFGF and TGF-β reduced the proliferation of BM-derived stromal cells by 25% [21]. Together, these data indicate that TKR signaling pathways play a pivotal role in HPL-induced stromal cell proliferation.

We further asked which central cellular key player involved in TKR signaling could mediate the HPL-derived growth signals. Our results showed that in response to HPL, the expression of STAT3, a major downstream effector of TKR signaling, was significantly enhanced in stromal cells. In addition, our data revealed that also the expression of MYC, a known strong promoter of cell proliferation [41,42,43] and direct transcriptional target gene of STAT3 [27,28,44], was enhanced in HPL-cultured stromal cells. In line with our results, enhanced mRNA expression of MYC was observed for WAT-derived stromal cells when cultured in human serum [45]. We also detected significantly elevated levels of phosphorylated STAT3 (Tyr705) and MYC (Ser62/Thr58) protein. These post-translational modifications are important for STAT3 and MYC protein stability, activation and the potential to induce cell cycle progression [46,47,48]. Therefore, our results indicated that functional STAT3 and MYC protein was expressed in response to HPL-culture conditions, thus pointing to an active role in supporting stromal cell proliferation in vitro.

**Figure 8:**
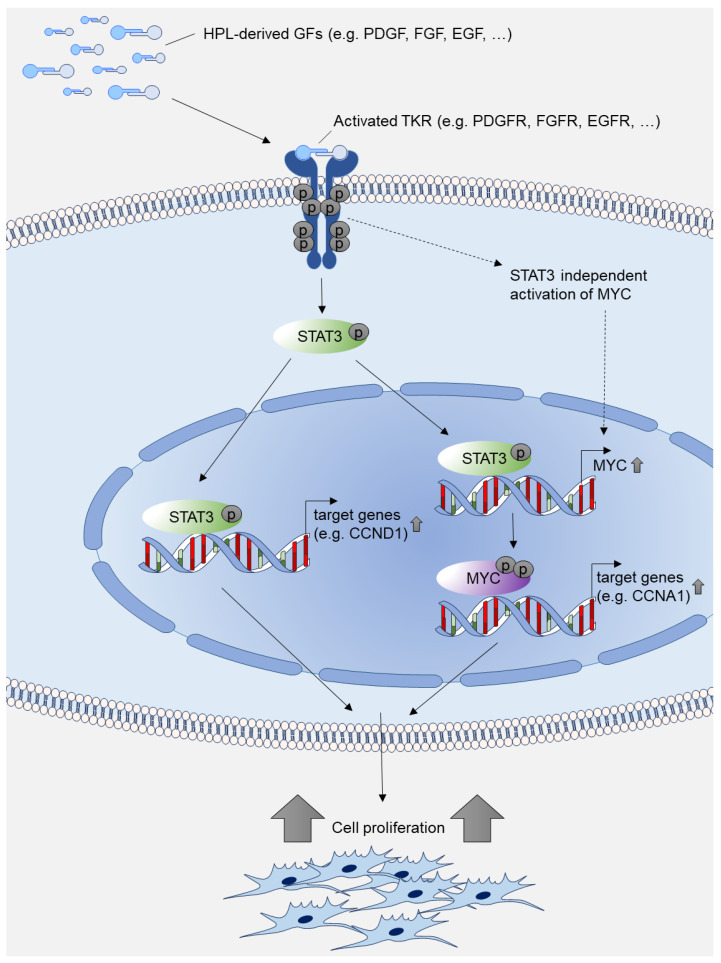
Model of HPL-mediated growth-promoting intracellular signal transduction. HPL-derived growth factors (GFs) such as PDGF, FGF and EGF activate tyrosine kinase receptors (TKRs) such as PDGFR, FGFR and EGFR. The activated signal results in increased expression of phosphorylated STAT3 and phosphorylated MYC, which further activate transcription of cell cycle target genes, thus promoting cell proliferation. Transcriptional activation of MYC may also be independent of STAT3 as described in [28,49,50].

Using the STAT3-specific inhibitors, STA21 and Stattic, we could significantly reduce stromal cell proliferation under HPL-based culture conditions. After treatment with STAT3-specific inhibitors, we could show that the expression of cell cycle-associated genes was significantly reduced. In a next step, we used increasing concentrations of HPL and observed a reversion of the STAT3-inhibitor effect. Thus, our data indicate that STAT3 is an essential key player in mediating the HPL-provided, growth-promoting signals. This effect may be conferred either directly by activating the transcription of cell cycle target genes such as CCND1 or indirectly by activating MYC, which induces the expression of genes that support cell proliferation (Figure 8).

Even though MYC is a direct transcriptional target gene of STAT3, it should be noted that PDGF may also activate MYC expression by STAT3-independent mechanisms. As shown by Chiariello et al., activated PDGFR stimulates MYC expression via Src/Rac in murine fibroblasts [28]. In another study, Iavarone et al. demonstrated that PDGF activates the MYC promoter by supporting the binding of specific AP-1 transcriptional activators [49]. Furthermore, FGF was shown to activate MYC expression directly through activated Akt/Erk signaling in a human breast cancer cell line [50]. Here, we showed that STAT3 is tightly involved in HPL-induced MYC gene regulation, as STAT3-specific inhibitors substantially reduced MYC expression in stromal cells.

Both STAT3 and MYC expression are described as being involved in tumor formation, cancer metabolism and even metastasis [51,52,53,54]. However, STAT3 overexpression in stromal cells does not necessarily mean tumorigenic transformation. Jiang et al. showed that overexpression of STAT3 in BM-derived stromal cells supports their differentiation into neural cells, which were applied to successfully treat spina bifida aperta in a rat model without observing tumor formation [55]. Two studies assessed the risk of tumorigenic transformation of human BM- and WAT-derived stromal cells when overexpressing MYC [41,45]; even though higher proliferation rates of MYC overexpressing stromal cells were observed, no signs for malignant transformation or teratoma growth were detected in mouse models. In addition, in vitro data also indicate no elevated risk for tumor transformation because of HPL-culture conditions. Several studies have shown that HPL-cultured stromal cells do not show higher numbers of colonies in colony formation assays compared to FBS culture [18,56,57].

Other pathways may contribute to mediate HPL’s growth-promoting effect as well. Next to ligands of TKR signaling receptors, HPL was shown to contain other growth-supporting factors, including matrix metalloproteinases (MMPs), tissue inhibitors of metalloproteinases (TIMP) and different chemokines such as chemokine (C-X-C Motif) ligand 4 (CXCL4) and ligand 12 (CXCL12) [13]. TIMPs, MMPs and the chemokines CXCL4 and CXCL12 are known to support stromal cell proliferation [58,59,60].

## 4. Materials and Methods

### 4.1. Ethical Statement

In this study, stromal cells from human bone marrow, umbilical cord and white adipose tissue were used. All donors of the UC and WAT tissue signed informed consent. The ethical committee of the Federal State of Salzburg, Austria appraised and waved the isolation of human stromal cells conducted as described in [61,62] (ethical vote numbers 415-E/1904/6-2015 and 415-E/1547/2-2012). BM samples were obtained from AllCells (Alameda, CA, USA). The work described was carried out in accordance with the 1964 Declaration of Helsinki and its later amendments. Samples were processed anonymously to protect privacy of each donor.

### 4.2. Isolation and Cultivation of Stromal Cells

The isolation of WAT-, UC- and BM-derived stromal cells (*n* = 3 for each tissue source) and their characterization were conducted as described previously [61,62]. For all experiments, only early cell passages 1–2 were used. Cells were cultured in alpha modified Minimum Essentials Eagle’s Medium (α-MEM, Sigma Aldrich, St. Louis, MO, USA) supplemented with 5.5 mM (N2)-L-alanyl-L-glutamine (Dipeptiven, Fresenius Kabi, Graz, Austria) and either with 10% FBS (FBS Premium; Biowest, Nuaille, France) or 10% HPL (production as described previously [19,20,56]) at 37 °C, 5% CO_2_ and 95% humidity. To avoid clot formation in HPL-based medium, 2 IU/mL heparin was added (Biochrom, Berlin, Germany).

### 4.3. RNA Isolation

Total RNA was isolated from stromal cells using High Pure RNA isolation kit (Roche Diagnostics, Rotkreuz, Switzerland) according to manufacturer’s instructions.

### 4.4. RT-qPCR

RT-qPCR analysis was performed using a LightCycler 480 II and LightCycler 480 SYBR Green I Master reagent (both Roche Diagnostics) according to manufacturer’s instructions. cDNA synthesis was carried out as described [63]. For normalization of sample material, human glyceraldehyde 3-phosphate dehydrogenase (GAPDH) expression was used. Data analysis was carried out as described in Regl et al. [64]. For RT-qPCR primer sequences, see Table S4.

### 4.5. Cell Cycle Antibody Array

To determine protein expression of cell cycle-specific genes, an ELISA-based antibody microarray (Full Moon BioSystems, Sunnyvale, CA, USA) was used according to manufacturer’s instructions. The customized antibody microarray contained 62 different cell cycle-specific antibodies and 2 antibodies directed against housekeeping genes spotted in technical quadruplicates. A list of all antibodies included is shown in Table S5. In brief, proteins from HPL- and FBS-cultured stromal cells isolated from WAT, UC and BM (*n* = 3 for each tissue source) were extracted using a non-denaturing extraction buffer (provided by the array manufacturer), that in addition contained a 1x protease inhibitor cocktail (Epigentek, Farmingdale, NY, USA). Protein lysate quality control and quantification was carried out using a NanoDrop UV spectrometer (ThermoFisher Scientific, Waltham, MA, USA) with λ = 280 nm. Only clear protein lysates with two well-separated peaks at 200–230 and 240–280 nm were subjected to further processing. After biotinylation, protein samples were conjugated to the pre-blocked antibody arrays. For detection, Cy3-conjugated streptavidin was applied. Dried arrays were scanned by Fullmoon’s Microarray Scanning Service (https://www.fullmoonbio.com/services/array-scanning/, accessed 15 June 2022). Array images were analyzed for signal intensity for each spot, average signal intensity for replicate spots, the coefficient of variation for replicate spots and fold change difference between HPL- and FBS-cultured stromal cell samples.

### 4.6. Inhibitor Treatment of Stromal Cells

Specific inhibitors of the tyrosine kinase receptors VEGFR/PDGFR (inhibitor: sunitinib, TargetMol, Wellesley Hills, MA, USA), EGFR (inhibitor: gefitinib; TargetMol) and VEGFR/FGFR (inhibitor: ponatinib, TargetMol) were applied to block the corresponding TKR-pathways. For each TKR-inhibitor, which were dissolved in dimethyl sulfoxide (DMSO, Wak-Chemie Medical GmbH, Steinbach, Germany), 1 µM was added to HPL-based growth medium. The STAT3 specific inhibitors Stattic, which blocks STAT3 phosphorylation, and STA21, which blocks STAT3 dimerization and nuclear translocation, were dissolved in DMSO and applied at the concentrations indicated. To determine the effect of each inhibitor on stromal cell proliferation, growth kinetics were compared to HPL-based growth medium only and DMSO-treated stromal cells.

### 4.7. Whole Genome Expression Analysis

A gene expression analysis comparing gene expression levels of stromal cells cultured in FBS- or HPL-based medium was conducted as described earlier [61]. In brief, isolated RNA was analyzed using an Agilent 2100 Bioanalyzer according to manufacturer’s protocol and finally was subjected for hybridization on an Affymetrix Human Gene 2.1 ST. For each tissue source, expression patterns of three individual donors were analyzed as described earlier using R/Rstudio (version 3.4.3, https://www.r-project.org)/Rstudio (https://www.rstudio.com, accessed on 29 September 2022, [61,65], Vienna, Austria). Genes with an adjusted *p*-value of ≤0.05 and an absolute fold change of ≥1.2 or ≤−1.2 were considered differentially expressed. Heatmaps were made using GraphPad Prism (version 9, GraphPad Software, San Diego, CA, USA).

### 4.8. xCELLigence Impedance Measurements

Proliferation was monitored using impedance measurements conducted on a xCELLigence RTCA MP instrument model W380 (ACEA Biosciences, San Diego, CA, USA). After incubating cell-free growth medium for 30 min at room temperature, the background impedance was determined. For each measurement, 1 × 10^3^ cells per well were seeded into 96-well e-plates (OLS Omni Life Science, Bremen, Germany). To allow cell attachment, e-plates were stored at room temperature for 30 min. The e-plates were then locked in the RTCA MP device, the impedance value of each well was automatically monitored by the xCELLigence system and expressed as normalized cell index value. All measurements were conducted in technical quadruplicates. Depending on the stromal cell tissue source and donor, cells were cultured between 70 and 145 h before further treatment, with monitoring every 30 min. After reaching a normalized cell index of at least 2, the e-plate was removed from the device and stromal cells were treated with different compounds as indicated by exchanging the growth medium with medium containing the compound. Finally, the measurement was continued by placing the e-plate back into the device, monitoring the proliferative behavior every 2 min for 4 h, followed by an observation every 15 min for up to 30 h. Quantitative analysis was carried out by calculation of doubling time and slope, which represents the rate of change of the normalized cell index over time, 24 h before and after treatment. All results were calculated by the instrument software (version 1.2.1.1002, ACEA Biosciences, San Diego, CA, USA).

### 4.9. SDS-Page and Western Blot Analysis

Equal volumes (15 µL) of protein lysates were separated on TGX Stain-Free precast gradient gels (4–20% Mini-PROTEANR TGX Stain-Free Protein Gels, Biorad, Munich, Germany) in Laemmli buffer. Proteins were then transferred to a polyvinylidene fluoride (PVDF) membrane (Biorad) and subsequently membranes were blocked for 2 h in 5% BSA in Tris buffered saline with 0.05% Tween 20 (TBST). Membranes were probed overnight at 4 °C in primary antibodies: STAT3 (124H6) mouse antibody; pSTAT3 (Tyr705) (D3A7) XPR rabbit antibody; MYC antibody; pMYC (Thr58) (E4Z2K) rabbit antibody; beta-Actin (8H10D10) mouse antibody (all 1:1000; Cell Signaling Technology, Danvers, MA, USA) in blocking solution. After washing membranes in TBST (3 × 15 min), membranes were probed with appropriate peroxidase-conjugated secondary antibodies (mouse anti-rabbit IgG (D4W3E) antibody and rabbit-anti-mouse IgG (D3V2A) antibody (both Cell Signaling Technology) in TBST for 1 h at room temperature. After final washing (3 × 15 min), blots were developed using a Chemidoc MP Imaging System and the Clarity Western ECL substrate (Biorad). Quantification of protein expression was carried out with ImageLab Software (Biorad) and normalized to the expression of beta-actin.

### 4.10. Statistical Analysis

All data shown are presented as mean ± SD of biological and technical replicates. Data were analyzed applying one-way or two-way ANOVA with Dunnett‘s multiple comparison tests using GraphPad Prism 9 (GraphPad Software, La Jolla, CA, USA) with *p* < 0.05 being considered as significant.

## 5. Conclusions

In conclusion, our study sheds light on the molecular mechanisms behind the growth-promoting effect of HPL. Even though the exact mechanisms of action of HPL are still not completely understood, the valuable functional effect of HPL can be most likely attributed to the HPL-borne mixture of pleiotropic growth factors. Here, we showed that these growth factors indeed activate the expression of genes promoting the cell cycle. We furthermore demonstrated that HPL’s growth-promoting effect could be assigned to the activation of STAT3 and MYC transcription factors. Although this might not be the sole mechanism, our findings contribute to the understanding of molecular interactions in HPL-based cell culture, it being of particular interest when stromal cells are propagated for therapeutic application.

## Data Availability

Not applicable.

## Supplementary Materials

The supporting information can be downloaded at: https://www.mdpi.com/article/10.3390/ijms232415782/s1.
