# Supplementary material for: Transcription Factors STAT3 and MYC Are Key Players of Human Platelet Lysate-Induced Cell Proliferation
Source: Int J Mol Sci. 2022 Dec 13;23(24):15782. doi: 10.3390/ijms232415782 (PMC9781157; doi:10.3390/ijms232415782)
Supplement: Supplementary file 1 [file ijms-23-15782-s001.zip › ijms-2054186-supplementary/Supplementary_material/Supplementary_table_S1.docx]

Supplementary table S1: Changes in mRNA and protein expression induced by HPL-based culture conditions compared to FBS-cultivation of BM-, UC- and WAT-derived stromal cells. Data shown are mean fold change (fc) values ± SD of three biological replicates measured in duplicates (qRT-PCR) or three biological replicates measured in quadruplicates (cell cycle antibody array). * p < 0.05, ** p < 0.01.

| Gene name | qRT-PCR (mRNA fc) | Antibody array (protein fc) | qRT-PCR (mRNA fc) | Antibody array (protein fc) | qRT-PCR (mRNA fc) | Antibody array (protein fc) |
| --- | --- | --- | --- | --- | --- | --- |
|  | ***BM*** | | ***UC*** | | ***WAT*** | |
| *ABL1* | 2.2 ± 1.4 | 1.1 ± 0.1 | 5.1 ± 3.5 * | 1.3 ± 0.1 * | 4.1 ± 1.0 ** | 1.4 ± 0.1 * |
| *APC2* | 7.5 ± 1.0 ** | 1.2 ± 0.1 | -1.4 ± 0.3 | -1.3 ± 0.1 * | 2.2 ± 0.7 * | 2.0 ± 0.3 * |
| *CCNA1* | 2.9 ± 1.2 * | 1.5 ± 0.2 * | 11.7 ± 3.0 ** | 3.5 ± 0.2 * | 8.6 ± 6.2 * | 1.8 ± 0.2 * |
| *CCNB1* | -3.8 ± 1.6* | 1.1 ± 0.1 | 1.0 ± 0.2 | 1.0 ± 0.1 | 2.3 ± 1.2 * | 1.6 ± 0.1 * |
| *CCND1* | 2.3 ± 0.8 * | 1.3 ± 0.1 * | 2.0 ± 0.2 * | 1.6 ± 0,1 * | -1.6 ± 1.2 | 1.1 ± 0.1 |
| *CCNE1* | -1.4 ± 0.4 | 1.3 ± 0.2 | -1.6 ± 0.5 | 0.8 ± 0.2 | 2.5 ± 1.6 | 1.9 ± 0.2 * |
| *CDK2* | 4.1 ± 1.5 * | 1.3 ± 0.2 | 2.1 ± 0.4 * | 1.3 ± 0.1 * | 1.3 ± 0.1 * | 1.2 ± 0.1 |
| *CDK3* | 1.3 ± 0.1 * | 1.3 ± 0.1 * | 2.1 ± 0.6 * | 1.3 ± 0.2 | 2.6 ± 1.6 | 2.4 ± 0.5 * |
| *CDKN1B – p27* | 3.7 ± 0.7 * | 1.0 ± 0.1 | 2.1 ± 0.6 * | 1.3 ± 0.1 * | 1.9 ± 0.9 | 1.0 ± 0.1 |
| *NUMA* | -1.6 ± 0.4 | 1.1 ± 0.1 | 6.4 ± 4.8 * | 2.2 ± 0.3 * | 5.2 ± 2.2 ** | 1.7 ± 0.2 * |
