# Supplementary material for: Transcription Factors STAT3 and MYC Are Key Players of Human Platelet Lysate-Induced Cell Proliferation
Source: Int J Mol Sci. 2022 Dec 13;23(24):15782. doi: 10.3390/ijms232415782 (PMC9781157; doi:10.3390/ijms232415782)
Supplement: Supplementary file 1 [file ijms-23-15782-s001.zip › ijms-2054186-supplementary/Supplementary_material/Supplementary_table_S2.docx]

Supplementary table S2: HPL-culture conditions result in differentially expressed protein levels (p < 0.05) of genes involved in cell cycle progression and control for BM-, UC- and WAT-derived stromal cells (indicated in the table as *all stromal cell types*) or in a tissue-source dependent manner (indicated in the table as *2 of 3 stromal cell types* and *one stromal cell type*).

| *Upregulated protein expression found in* | *Stromal cell type* | *Total # of proteins upregulated* | *Protein detected (p < 0.05)* |
| --- | --- | --- | --- |
| all stromal cell types | BM UC WAT | 10 | Cyclin A1 (C-term) Cullin-3 (CUL-3) Cdk3 Glycogen Synthase Kinase 3b (GSK3b) p19ARF Beta actin Cyclin A(A1/A2) (inter) p73a Cdk1/p34cdc2 14.3.3, Pan |
| 2 of 3 stromal cell types | BM UC | 6 | E2F-1 Chk1 CDC14A Retinoblastoma (Rb) (Phospho-specific Serine 608) RAD 51 p57Kip2 Cyclin D1 |
|  | UC WAT | 6 | p73 Cdk2 Ki67 p35nck5a NuMA c-Abl |
|  | BM WAT | 6 | Cdk8 Cdk7 Cyclin E APC2 APC11 CDC37 |
| one stromal cell type | UC | 10 | Cdk4 p21WAF1 E2F-3 CDC6 p130cas p130 CDC25C ATM p27Kip1 |
|  | BM | 2 | Cyclin E2 TOPO II beta |
|  | WAT | 11 | PCNA Cyclin B1 CDC34 p19Skp1 CDC47 p16INK4a p18INK4c Tubulin-b p130 (RBL2) Cullin-2 (CUL-2) Cyclin C |
| *Downregulated protein expression found in* | *Stromal cell type* | *Total # of proteins downregulated* | *Proteins* |
| all stromal cell types | BM UC WAT | 1 | Mitochondria Ab-2 |
| one stromal cell type | UC | 6 | PCNA Retinoblastoma p53 Cyclin E Tubulin-b APC2 |
|  | WAT | 5 | RAD 51 Chk1 ATM E2F-1 p130cas |
