# Supplementary material for: Transcription Factors STAT3 and MYC Are Key Players of Human Platelet Lysate-Induced Cell Proliferation
Source: Int J Mol Sci. 2022 Dec 13;23(24):15782. doi: 10.3390/ijms232415782 (PMC9781157; doi:10.3390/ijms232415782)
Supplement: Supplementary file 1 [file ijms-23-15782-s001.zip › ijms-2054186-supplementary/Supplementary_material/Supplementary_table_S3.docx]

Supplementary table S3: Upregulation of cell cycle target genes by HPL-based culture conditions (≥ 1.2 fold) for each stromal cell type ranked from highest to lowest (FC = fold change, for all data shown p < 0.05).

| Stromal cell type | *#* | *Protein name* | *Function according to genecards data base entry* | *FC* |
| --- | --- | --- | --- | --- |
| **BM** | 1 | 14.3.3, Pan | 14-3-3 proteins are a family of small, widely expressed, highly conserved cytosolic proteins. 14-3-3 proteins bind to and influence the activities of a diverse group of molecules involved in signal transduction, cell cycle regulation and apoptosis, including Raf, PKC, Bad, Cbl, and c-Bcr. Interactions between 14-3-3 and target proteins are strongly influenced by the phosphorylation state of 14-3-3 and the target protein. | 2,9 |
|  | 2 | p73a | Tumor Protein P73; encodes a member of the p53 family of transcription factors involved in cellular responses to stress and development. It maps to a region on chromosome 1p36 that is frequently deleted in neuroblastoma and other tumors, and thought to contain multiple tumor suppressor genes. The demonstration that this gene is monoallelically expressed (likely from the maternal allele), supports the notion that it is a candidate gene for neuroblastoma. Many transcript variants resulting from alternative splicing and/or use of alternate promoters have been found for this gene, but the biological validity and the full-length nature of some variants have not been determined. | 2,8 |
|  | 3 | Chk1 | CHEK1; Checkpoint Kinase 1; gene belongs to the Ser/Thr protein kinase family. It is required for checkpoint mediated cell cycle arrest in response to DNA damage or the presence of unreplicated DNA. This protein acts to integrate signals from ATM and ATR, two cell cycle proteins involved in DNA damage responses, that also associate with chromatin in meiotic prophase I. Phosphorylation of CDC25A protein phosphatase by this protein is required for cells to delay cell cycle progression in response to double-strand DNA breaks. | 2,3 |
|  | 4 | p19ARF | Cyclin Dependent Kinase Inhibitor 2A, CDKN2A; This ARF product functions as a stabilizer of the tumor suppressor protein p53 as it can interact with, and sequester, the E3 ubiquitin-protein ligase MDM2, a protein responsible for the degradation of p53. In | 2,2 |
|  | 5 | E2F-1 | E2F Transcription Factor 1; member of the E2F family of transcription factors. The E2F family plays a crucial role in the control of cell cycle and action of tumor suppressor proteins and is also a target of the transforming proteins of small DNA tumor viruses. The E2F proteins contain several evolutionally conserved domains found in most members of the family. These domains include a DNA binding domain, a dimerization domain which determines interaction with the differentiation regulated transcription factor proteins (DP), a transactivation domain enriched in acidic amino acids, and a tumor suppressor protein association domain which is embedded within the transactivation domain. This protein and another 2 members, E2F2 and E2F3, have an additional cyclin binding domain. This protein binds preferentially to retinoblastoma protein pRB in a cell-cycle dependent manner. It can mediate both cell proliferation and p53-dependent/independent apoptosis. | 2,2 |
|  | 6 | Cullin-3 (CUL-3) | Cullin 3; encodes a member of the cullin protein family. The encoded protein plays a critical role in the polyubiquitination and subsequent degradation of specific protein substrates as the core component and scaffold protein of an E3 ubiquitin ligase complex. Complexes including the encoded protein may also play a role in late endosome maturation. | 2,0 |
|  | 7 | Beta actin | ACTB; Actins are highly conserved proteins that are involved in cell motility, structure, integrity, and intercellular signaling; n addition to their role in the cytoplasmic cytoskeleton, G- and F-actin also localize in the nucleus, and regulate gene transcription and motility and repair of damaged DNA | 2,0 |
|  | 8 | Glycogen Synthase Kinase 3b (GSK3b) | Glycogen Synthase Kinase 3 Beta; It is a negative regulator of glucose homeostasis and is involved in energy metabolism, inflammation, ER-stress, mitochondrial dysfunction, and apoptotic pathways. | 1,7 |
|  | 9 | Cdk8 | Cyclin Dependent Kinase 8; member of the cyclin-dependent protein kinase (CDK) family. CDK family members are known to be important regulators of cell cycle progression. This kinase and its regulatory subunit, cyclin C, are components of the Mediator transcriptional regulatory complex, involved in both transcriptional activation and repression by phosphorylation of the carboxy-terminal domain of the largest subunit of RNA polymerase II. This kinase regulates transcription by targeting the cyclin-dependent kinase 7 subunits of the general transcription initiation factor IIH, thus providing a link between the Mediator complex and the basal transcription machinery. Multiple pseudogenes of this gene have been identified. Alternative splicing results in multiple transcript variants. | 1,5 |
|  | 10 | Cyclin A1 (C-term) | Cyclin A1; encoded by this gene belongs to the highly conserved cyclin family, whose members are characterized by a dramatic periodicity in protein abundance through the cell cycle. Cyclins function as regulators of CDK kinases. Different cyclins exhibit distinct expression and degradation patterns which contribute to the temporal coordination of each mitotic event. The cyclin encoded by this gene was shown to be expressed in testis and brain, as well as in several leukemic cell lines, and is thought to primarily function in the control of the germline meiotic cell cycle. This cyclin binds both CDK2 and CDC2 kinases, which give two distinct kinase activities, one appearing in S phase, the other in G2, and thus regulate separate functions in cell cycle. This cyclin was found to bind to important cell cycle regulators, such as Rb family proteins, transcription factor E2F-1, and the p21 family proteins. | 1,5 |
|  | 11 | Cdk3 | Cyclin Dependent Kinase 3; The protein promotes entry into S phase, in part by activating members of the E2F family of transcription factors. The protein also associates with cyclin C and phosphorylates the retinoblastoma 1 protein to promote exit from G0. | 1,3 |
|  | 12 | Cyclin E2 | Cyclin E2; belongs to the highly conserved cyclin family, whose members are characterized by a dramatic periodicity in protein abundance through the cell cycle. Cyclins function as regulators of CDK kinases. Different cyclins exhibit distinct expression and degradation patterns which contribute to the temporal coordination of each mitotic event. This cyclin forms a complex with and functions as a regulatory subunit of CDK2. This cyclin has been shown to specifically interact with CIP/KIP family of CDK inhibitors, and plays a role in cell cycle G1/S transition. The expression of this gene peaks at the G1-S phase and exhibits a pattern of tissue specificity distinct from that of cyclin E1. A significantly increased expression level of this gene was observed in tumor-derived cells. | 1,3 |
|  | 13 | RAD 51 | RAD51 Recombinase; encoded by this gene is a member of the RAD51 protein family. RAD51 family members are highly similar to bacterial RecA and Saccharomyces cerevisiae Rad51, and are known to be involved in the homologous recombination and repair of DNA. This protein can interact with the ssDNA-binding protein RPA and RAD52, and it is thought to play roles in homologous pairing and strand transfer of DNA. This protein is also found to interact with BRCA1 and BRCA2, which may be important for the cellular response to DNA damage. BRCA2 is shown to regulate both the intracellular localization and DNA-binding ability of this protein. Loss of these controls following BRCA2 inactivation may be a key event leading to genomic instability and tumorigenesis. | 1,3 |
|  | 14 | GAPDH | Glyceraldehyde-3-Phosphate Dehydrogenase; member of the glyceraldehyde-3-phosphate dehydrogenase protein family. The encoded protein has been identified as a moonlighting protein based on its ability to perform mechanistically distinct functions. The product of this gene catalyzes an important energy-yielding step in carbohydrate metabolism, the reversible oxidative phosphorylation of glyceraldehyde-3-phosphate in the presence of inorganic phosphate and nicotinamide adenine dinucleotide (NAD). The encoded protein has additionally been identified to have uracil DNA glycosylase activity in the nucleus. Also, this protein contains a peptide that has antimicrobial activity against E. coli, P. aeruginosa, and C. albicans. Studies of a similar protein in mouse have assigned a variety of additional functions including nitrosylation of nuclear proteins, the regulation of mRNA stability, and acting as a transferrin receptor on the cell surface of macrophage. | 1,3 |
|  | 15 | Cyclin A(A1/A2) (inter) | Cyclin A1; encoded by this gene belongs to the highly conserved cyclin family, whose members are characterized by a dramatic periodicity in protein abundance through the cell cycle. Cyclins function as regulators of CDK kinases. Different cyclins exhibit distinct expression and degradation patterns which contribute to the temporal coordination of each mitotic event. The cyclin encoded by this gene was shown to be expressed in testis and brain, as well as in several leukemic cell lines, and is thought to primarily function in the control of the germline meiotic cell cycle. This cyclin binds both CDK2 and CDC2 kinases, which give two distinct kinase activities, one appearing in S phase, the other in G2, and thus regulate separate functions in cell cycle. This cyclin was found to bind to important cell cycle regulators, such as Rb family proteins, transcription factor E2F-1, and the p21 family proteins. | 1,3 |
|  | 16 | Cyclin E | Cyclin E1; belongs to the highly conserved cyclin family, whose members are characterized by a dramatic periodicity in protein abundance through the cell cycle. Cyclins function as regulators of CDK kinases. Different cyclins exhibit distinct expression and degradation patterns which contribute to the temporal coordination of each mitotic event. This cyclin forms a complex with and functions as a regulatory subunit of CDK2, whose activity is required for cell cycle G1/S transition. This protein accumulates at the G1-S phase boundary and is degraded as cells progress through S phase. Overexpression of this gene has been observed in many tumors, which results in chromosome instability, and thus may contribute to tumorigenesis. This protein was found to associate with, and be involved in, the phosphorylation of NPAT protein (nuclear protein mapped to the ATM locus), which participates in cell-cycle regulated histone gene expression and plays a critical role in promoting cell-cycle progression in the absence of pRB. | 1,3 |
|  | 17 | Cdk7 | Cyclin Dependent Kinase 7; member of the cyclin-dependent protein kinase (CDK) family. CDK family members are highly similar to the gene products of Saccharomyces cerevisiae cdc28, and Schizosaccharomyces pombe cdc2, and are known to be important regulators of cell cycle progression. This protein forms a trimeric complex with cyclin H and MAT1, which functions as a Cdk-activating kinase (CAK). It is an essential component of the transcription factor TFIIH, that is involved in transcription initiation and DNA repair. This protein is thought to serve as a direct link between the regulation of transcription and the cell cycle. | 1,2 |
|  | 18 | CDC37 | Cell Division Cycle 37, HSP90 Cochaperone; highly similar to Cdc 37, a cell division cycle control protein of Sacchromyces cerevisiae. This protein is a molecular chaperone with specific function in cell signal transduction. It has been shown to form complex with Hsp90 and a variety of protein kinases including CDK4, CDK6, SRC, RAF-1, MOK, as well as eIF2 alpha kinases. It is thought to play a critical role in directing Hsp90 to its target kinases. | 1,2 |
|  | 19 | APC2 | ANAPC2; Anaphase Promoting Complex Subunit 2; A large protein complex, termed the anaphase-promoting complex (APC), or the cyclosome, promotes metaphase-anaphase transition by ubiquitinating its specific substrates such as mitotic cyclins and anaphase inhibitor, which are subsequently degraded by the 26S proteasome. Biochemical studies have shown that the vertebrate APC contains eight subunits. | 1,2 |
|  | 20 | TOPO II beta | Topo IIBeta plays important roles in synthesis and transcription of DNA as well as chromosomal segregation during mitosis. It is present ubiquitously in normal cells and is upregulated in tumors and proliferating cells. Topo IIBeta is also implicated in drug resistance of tumor cells. | 1,2 |
|  | 21 | Cdk1/p34cdc2 | Cyclin Dependent Kinase 1; protein encoded by this gene is a member of the Ser/Thr protein kinase family. This protein is a catalytic subunit of the highly conserved protein kinase complex known as M-phase promoting factor (MPF), which is essential for G1/S and G2/M phase transitions of eukaryotic cell cycle. Mitotic cyclins stably associate with this protein and function as regulatory subunits. The kinase activity of this protein is controlled by cyclin accumulation and destruction through the cell cycle. The phosphorylation and dephosphorylation of this protein also play important regulatory roles in cell cycle control. | 1,2 |
|  | 22 | APC11 | ANAPC11; Anaphase Promoting Complex Subunit 11; cullin protein ANAPC2, constitutes the catalytic component of the anaphase promoting complex/cyclosome (APC/C), a cell cycle-regulated E3 ubiquitin ligase that controls progression through mitosis and the G1 phase of the cell cycle. | 1,2 |
|  | 23 | Retinoblastoma (Rb) (Phospho-specific Serine 608) | RB Transcriptional Corepressor 1; Retinoblastoma 1; encoded by this gene is a negative regulator of the cell cycle and was the first tumor suppressor gene found. The encoded protein also stabilizes constitutive heterochromatin to maintain the overall chromatin structure. The active, hypophosphorylated form of the protein binds transcription factor E2F1; Key regulator of entry into cell division that acts as a tumor suppressor. Promotes G0-G1 transition when phosphorylated by CDK3/cyclin-C. Acts as a transcription repressor of E2F1 target genes. | 1,2 |
|  | 24 | CDC14A Phosphatase | Cell Division Cycle 14A; This protein has been shown to interact with, and dephosphorylate tumor suppressor protein p53, and is thought to regulate the function of p53; It is highly similar to Saccharomyces cerevisiae Cdc14, a protein tyrosine phosphatase involved in the exit of cell mitosis and initiation of DNA replication, suggesting a role in cell cycle control. | 1,2 |
| **UC** | 1 | p73a | Tumor Protein P73; encodes a member of the p53 family of transcription factors involved in cellular responses to stress and development. It maps to a region on chromosome 1p36 that is frequently deleted in neuroblastoma and other tumors, and thought to contain multiple tumor suppressor genes. The demonstration that this gene is monoallelically expressed (likely from the maternal allele), supports the notion that it is a candidate gene for neuroblastoma. Many transcript variants resulting from alternative splicing and/or use of alternate promoters have been found for this gene, but the biological validity and the full-length nature of some variants have not been determined. | 5,4 |
|  | 2 | p19ARF | Cyclin Dependent Kinase Inhibitor 2A, CDKN2A; This ARF product functions as a stabilizer of the tumor suppressor protein p53 as it can interact with, and sequester, the E3 ubiquitin-protein ligase MDM2, a protein responsible for the degradation of p53. In | 5,0 |
|  | 3 | E2F-1 | E2F Transcription Factor 1; member of the E2F family of transcription factors. The E2F family plays a crucial role in the control of cell cycle and action of tumor suppressor proteins and is also a target of the transforming proteins of small DNA tumor viruses. The E2F proteins contain several evolutionally conserved domains found in most members of the family. These domains include a DNA binding domain, a dimerization domain which determines interaction with the differentiation regulated transcription factor proteins (DP), a transactivation domain enriched in acidic amino acids, and a tumor suppressor protein association domain which is embedded within the transactivation domain. This protein and another 2 members, E2F2 and E2F3, have an additional cyclin binding domain. This protein binds preferentially to retinoblastoma protein pRB in a cell-cycle dependent manner. It can mediate both cell proliferation and p53-dependent/independent apoptosis. | 4,6 |
|  | 4 | RAD 51 | RAD51 Recombinase; encoded by this gene is a member of the RAD51 protein family. RAD51 family members are highly similar to bacterial RecA and Saccharomyces cerevisiae Rad51, and are known to be involved in the homologous recombination and repair of DNA. This protein can interact with the ssDNA-binding protein RPA and RAD52, and it is thought to play roles in homologous pairing and strand transfer of DNA. This protein is also found to interact with BRCA1 and BRCA2, which may be important for the cellular response to DNA damage. BRCA2 is shown to regulate both the intracellular localization and DNA-binding ability of this protein. Loss of these controls following BRCA2 inactivation may be a key event leading to genomic instability and tumorigenesis. | 3,7 |
|  | 5 | Cyclin A1 (C-term) | Cyclin A1; encoded by this gene belongs to the highly conserved cyclin family, whose members are characterized by a dramatic periodicity in protein abundance through the cell cycle. Cyclins function as regulators of CDK kinases. Different cyclins exhibit distinct expression and degradation patterns which contribute to the temporal coordination of each mitotic event. The cyclin encoded by this gene was shown to be expressed in testis and brain, as well as in several leukemic cell lines, and is thought to primarily function in the control of the germline meiotic cell cycle. This cyclin binds both CDK2 and CDC2 kinases, which give two distinct kinase activities, one appearing in S phase, the other in G2, and thus regulate separate functions in cell cycle. This cyclin was found to bind to important cell cycle regulators, such as Rb family proteins, transcription factor E2F-1, and the p21 family proteins. | 3,5 |
|  | 6 | Cullin-3 (CUL-3) | Cullin 3; encodes a member of the cullin protein family. The encoded protein plays a critical role in the polyubiquitination and subsequent degradation of specific protein substrates as the core component and scaffold protein of an E3 ubiquitin ligase complex. Complexes including the encoded protein may also play a role in late endosome maturation. | 3,1 |
|  | 7 | p73 | Tumor Protein P73; encodes a member of the p53 family of transcription factors involved in cellular responses to stress and development. It maps to a region on chromosome 1p36 that is frequently deleted in neuroblastoma and other tumors, and thought to contain multiple tumor suppressor genes. The demonstration that this gene is monoallelically expressed (likely from the maternal allele), supports the notion that it is a candidate gene for neuroblastoma. Many transcript variants resulting from alternative splicing and/or use of alternate promoters have been found for this gene, but the biological validity and the full-length nature of some variants have not been determined. | 2,8 |
|  | 8 | Chk1 | CHEK1; Checkpoint Kinase 1; gene belongs to the Ser/Thr protein kinase family. It is required for checkpoint mediated cell cycle arrest in response to DNA damage or the presence of unreplicated DNA. This protein acts to integrate signals from ATM and ATR, two cell cycle proteins involved in DNA damage responses, that also associate with chromatin in meiotic prophase I. Phosphorylation of CDC25A protein phosphatase by this protein is required for cells to delay cell cycle progression in response to double-strand DNA breaks. | 2,8 |
|  | 9 | Cyclin A(A1/A2) (inter) | Cyclin A1; encoded by this gene belongs to the highly conserved cyclin family, whose members are characterized by a dramatic periodicity in protein abundance through the cell cycle. Cyclins function as regulators of CDK kinases. Different cyclins exhibit distinct expression and degradation patterns which contribute to the temporal coordination of each mitotic event. The cyclin encoded by this gene was shown to be expressed in testis and brain, as well as in several leukemic cell lines, and is thought to primarily function in the control of the germline meiotic cell cycle. This cyclin binds both CDK2 and CDC2 kinases, which give two distinct kinase activities, one appearing in S phase, the other in G2, and thus regulate separate functions in cell cycle. This cyclin was found to bind to important cell cycle regulators, such as Rb family proteins, transcription factor E2F-1, and the p21 family proteins. | 2,6 |
|  | 10 | NuMA | Nuclear Mitotic Apparatus Protein 1; a large protein that forms a structural component of the nuclear matrix. The encoded protein interacts with microtubules and plays a role in the formation and organization of the mitotic spindle during cell division. | 2,2 |
|  | 11 | Cdk4 | Cyclin Dependent Kinase 4; catalytic subunit of the protein kinase complex that is important for cell cycle G1 phase progression. The activity of this kinase is restricted to the G1-S phase, which is controlled by the regulatory subunits D-type cyclins and CDK inhibitor p16(INK4a). This kinase was shown to be responsible for the phosphorylation of retinoblastoma gene product (Rb). Mutations in this gene as well as in its related proteins including D-type cyclins, p16(INK4a) and Rb were all found to be associated with tumorigenesis of a variety of cancers. | 2,2 |
|  | 12 | Beta actin | ACTB; Actins are highly conserved proteins that are involved in cell motility, structure, integrity, and intercellular signaling; n addition to their role in the cytoplasmic cytoskeleton, G- and F-actin also localize in the nucleus, and regulate gene transcription and motility and repair of damaged DNA | 2,2 |
|  | 13 | GAPDH | Glyceraldehyde-3-Phosphate Dehydrogenase; member of the glyceraldehyde-3-phosphate dehydrogenase protein family. The encoded protein has been identified as a moonlighting protein based on its ability to perform mechanistically distinct functions. The product of this gene catalyzes an important energy-yielding step in carbohydrate metabolism, the reversible oxidative phosphorylation of glyceraldehyde-3-phosphate in the presence of inorganic phosphate and nicotinamide adenine dinucleotide (NAD). The encoded protein has additionally been identified to have uracil DNA glycosylase activity in the nucleus. Also, this protein contains a peptide that has antimicrobial activity against E. coli, P. aeruginosa, and C. albicans. Studies of a similar protein in mouse have assigned a variety of additional functions including nitrosylation of nuclear proteins, the regulation of mRNA stability, and acting as a transferrin receptor on the cell surface of macrophage. | 2,1 |
|  | 14 | Glycogen Synthase Kinase 3b (GSK3b) | Glycogen Synthase Kinase 3 Beta; It is a negative regulator of glucose homeostasis and is involved in energy metabolism, inflammation, ER-stress, mitochondrial dysfunction, and apoptotic pathways. | 2,1 |
|  | 15 | CDC14A Phosphatase | Cell Division Cycle 14A; This protein has been shown to interact with, and dephosphorylate tumor suppressor protein p53, and is thought to regulate the function of p53; It is highly similar to Saccharomyces cerevisiae Cdc14, a protein tyrosine phosphatase involved in the exit of cell mitosis and initiation of DNA replication, suggesting a role in cell cycle control. | 2,0 |
|  | 16 | CDC6 | Cell Division Cycle 6; a protein essential for the initiation of DNA replication. This protein functions as a regulator at the early steps of DNA replication. It localizes in cell nucleus during cell cyle G1, but translocates to the cytoplasm at the start of S phase. The subcellular translocation of this protein during cell cyle is regulated through its phosphorylation by Cdks. Transcription of this protein was reported to be regulated in response to mitogenic signals through transcriptional control mechanism involving E2F proteins. | 2,0 |
|  | 17 | Retinoblastoma (Rb) (Phospho-specific Serine 608) | RB Transcriptional Corepressor 1; Retinoblastoma 1; encoded by this gene is a negative regulator of the cell cycle and was the first tumor suppressor gene found. The encoded protein also stabilizes constitutive heterochromatin to maintain the overall chromatin structure. The active, hypophosphorylated form of the protein binds transcription factor E2F1; Key regulator of entry into cell division that acts as a tumor suppressor. Promotes G0-G1 transition when phosphorylated by CDK3/cyclin-C. Acts as a transcription repressor of E2F1 target genes. | 1,9 |
|  | 18 | p130 | RB Transcriptional Corepressor Like 2; Key regulator of entry into cell division. Directly involved in heterochromatin formation by maintaining overall chromatin structure and, in particular, that of constitutive heterochromatin by stabilizing histone methylation. Recruits and targets histone methyltransferases KMT5B and KMT5C, leading to epigenetic transcriptional repression. | 1,6 |
|  | 19 | ATM | ATM Serine/Threonine Kinase; is an important cell cycle checkpoint kinase that phosphorylates; thus, it functions as a regulator of a wide variety of downstream proteins, including tumor suppressor proteins p53 and BRCA1, checkpoint kinase CHK2, checkpoint proteins RAD17 and RAD9, and DNA repair protein NBS1. This protein and the closely related kinase ATR are thought to be master controllers of cell cycle checkpoint signaling pathways that are required for cell response to DNA damage and for genome stability. | 1,6 |
|  | 20 | Cyclin D1 | Cyclin D1; belongs to the highly conserved cyclin family, whose members are characterized by a dramatic periodicity in protein abundance throughout the cell cycle. Cyclins function as regulators of CDK kinases. Different cyclins exhibit distinct expression and degradation patterns which contribute to the temporal coordination of each mitotic event. This cyclin forms a complex with and functions as a regulatory subunit of CDK4 or CDK6, whose activity is required for cell cycle G1/S transition. This protein has been shown to interact with tumor suppressor protein Rb and the expression of this gene is regulated positively by Rb. | 1,6 |
|  | 21 | 14.3.3, Pan | 14-3-3 proteins are a family of small, widely expressed, highly conserved cytosolic proteins. 14-3-3 proteins bind to and influence the activities of a diverse group of molecules involved in signal transduction, cell cycle regulation and apoptosis, including Raf, PKC, Bad, Cbl, and c-Bcr. Interactions between 14-3-3 and target proteins are strongly influenced by the phosphorylation state of 14-3-3 and the target protein. | 1,5 |
|  | 22 | Ki67 | Marker Of Proliferation Ki-67; Required to maintain individual mitotic chromosomes dispersed in the cytoplasm following nuclear envelope disassembly (PubMed:27362226). Associates with the surface of the mitotic chromosome, the perichromosomal layer, and covers a substantial fraction of the chromosome surface (PubMed:27362226). Prevents chromosomes from collapsing into a single chromatin mass by forming a steric and electrostatic charge barrier: the protein has a high net electrical charge and acts as a surfactant, dispersing chromosomes and enabling independent chromosome motility (PubMed:27362226). Binds DNA, with a preference for supercoiled DNA and AT-rich DNA (PubMed:10878551). | 1,5 |
|  | 23 | p57Kip2 | Cyclin Dependent Kinase Inhibitor 1C, CDKN1C; strong inhibitor of several G1 cyclin/Cdk complexes and a negative regulator of cell proliferation. | 1,4 |
|  | 24 | Cdk1/p34cdc2 | Cyclin Dependent Kinase 1; protein encoded by this gene is a member of the Ser/Thr protein kinase family. This protein is a catalytic subunit of the highly conserved protein kinase complex known as M-phase promoting factor (MPF), which is essential for G1/S and G2/M phase transitions of eukaryotic cell cycle. Mitotic cyclins stably associate with this protein and function as regulatory subunits. The kinase activity of this protein is controlled by cyclin accumulation and destruction through the cell cycle. The phosphorylation and dephosphorylation of this protein also play important regulatory roles in cell cycle control. | 1,4 |
|  | 25 | c-Abl | ABL Proto-Oncogene 1; Abelson Tyrosine-Protein Kinase 1; protein tyrosine kinase involved in a variety of cellular processes, including cell division, adhesion, differentiation, and response to stress. | 1,3 |
|  | 26 | E2F-3 | E2F Transcription Factor 3; member of a small family of transcription factors that function through binding of DP interaction partner proteins. The encoded protein recognizes a specific sequence motif in DNA and interacts directly with the retinoblastoma protein (pRB) to regulate the expression of genes involved in the cell cycle. | 1,3 |
|  | 27 | Cdk2 | Cyclin Dependent Kinase 2; protein is the catalytic subunit of the cyclin-dependent protein kinase complex, which regulates progression through the cell cycle. Activity of this protein is especially critical during the G1 to S phase transition. This protein associates with and regulated by other subunits of the complex including cyclin A or E, CDK inhibitor p21Cip1 (CDKN1A), and p27Kip1 (CDKN1B). | 1,3 |
|  | 28 | p27Kip1 | Cyclin Dependent Kinase Inhibitor 1B, CDKN1B; encodes a cyclin-dependent kinase inhibitor, which shares a limited similarity with CDK inhibitor CDKN1A/p21. The encoded protein binds to and prevents the activation of cyclin E-CDK2 or cyclin D-CDK4 complexes, and thus controls the cell cycle progression at G1. The degradation of this protein, which is triggered by its CDK dependent phosphorylation and subsequent ubiquitination by SCF complexes, is required for the cellular transition from quiescence to the proliferative state. | 1,3 |
|  | 29 | p35nck5a | Cyclin Dependent Kinase 5 Regulatory Subunit 1; encoded by this gene (p35) is a neuron-specific activator of cyclin-dependent kinase 5 (CDK5); the activation of CDK5 is required for proper development of the central nervous system. The p35 form of this protein is proteolytically cleaved by calpain, generating a p25 form. The cleavage of p35 into p25 results in relocalization of the protein from the cell periphery to nuclear and perinuclear regions. P25 deregulates CDK5 activity by prolonging its activation and changing its cellular location. The p25 form accumulates in the brain neurons of patients with Alzheimer's disease. This accumulation correlates with an increase in CDK5 kinase activity, and may lead to aberrantly phosphorylated forms of the microtubule-associated protein tau, which contributes to Alzheimer's disease. | 1,2 |
|  | 30 | Cdk3 | Cyclin Dependent Kinase 3; The protein promotes entry into S phase, in part by activating members of the E2F family of transcription factors. The protein also associates with cyclin C and phosphorylates the retinoblastoma 1 protein to promote exit from G0. | 1,2 |
|  | 31 | p21WAF1 | Cyclin Dependent Kinase Inhibitor 1A, CDKN1A; encodes a potent cyclin-dependent kinase inhibitor. The encoded protein binds to and inhibits the activity of cyclin-cyclin-dependent kinase2 or -cyclin-dependent kinase4 complexes, and thus functions as a regulator of cell cycle progression at G1. The expression of this gene is tightly controlled by the tumor suppressor protein p53, through which this protein mediates the p53-dependent cell cycle G1 phase arrest in response to a variety of stress stimuli. This protein can interact with proliferating cell nuclear antigen, a DNA polymerase accessory factor, and plays a regulatory role in S phase DNA replication and DNA damage repair. This protein was reported to be specifically cleaved by CASP3-like caspases, which thus leads to a dramatic activation of cyclin-dependent kinase2, and may be instrumental in the execution of apoptosis following caspase activation. | 1,2 |
|  | 32 | p130cas | BCAR1 Scaffold Protein, Cas Family Member; member of the Crk-associated substrate (CAS) family of scaffold proteins, characterized by the presence of multiple protein-protein interaction domains and many serine and tyrosine phosphorylation sites. The encoded protein contains a Src-homology 3 (SH3) domain, a proline-rich domain, a substrate domain which contains 15 repeat of the YxxP consensus phosphorylation motif for Src family kinases, a serine-rich domain, and a bipartite Src-binding domain, which can bind both SH2 and SH3 domains. This adaptor protein functions in multiple cellular pathways, including in cell motility, apoptosis and cell cycle control. | 1,2 |
| **WAT** | 1 | Cdk3 | Cyclin Dependent Kinase 3; The protein promotes entry into S phase, in part by activating members of the E2F family of transcription factors. The protein also associates with cyclin C and phosphorylates the retinoblastoma 1 protein to promote exit from G0. | 2,4 |
|  | 2 | APC2 | ANAPC2; Anaphase Promoting Complex Subunit 2; A large protein complex, termed the anaphase-promoting complex (APC), or the cyclosome, promotes metaphase-anaphase transition by ubiquitinating its specific substrates such as mitotic cyclins and anaphase inhibitor, which are subsequently degraded by the 26S proteasome. Biochemical studies have shown that the vertebrate APC contains eight subunits. | 2,0 |
|  | 3 | Cyclin E | Cyclin E1; belongs to the highly conserved cyclin family, whose members are characterized by a dramatic periodicity in protein abundance through the cell cycle. Cyclins function as regulators of CDK kinases. Different cyclins exhibit distinct expression and degradation patterns which contribute to the temporal coordination of each mitotic event. This cyclin forms a complex with and functions as a regulatory subunit of CDK2, whose activity is required for cell cycle G1/S transition. This protein accumulates at the G1-S phase boundary and is degraded as cells progress through S phase. Overexpression of this gene has been observed in many tumors, which results in chromosome instability, and thus may contribute to tumorigenesis. This protein was found to associate with, and be involved in, the phosphorylation of NPAT protein (nuclear protein mapped to the ATM locus), which participates in cell-cycle regulated histone gene expression and plays a critical role in promoting cell-cycle progression in the absence of pRB. | 1,9 |
|  | 4 | Cullin-3 (CUL-3) | Cullin 3; encodes a member of the cullin protein family. The encoded protein plays a critical role in the polyubiquitination and subsequent degradation of specific protein substrates as the core component and scaffold protein of an E3 ubiquitin ligase complex. Complexes including the encoded protein may also play a role in late endosome maturation. | 1,9 |
|  | 5 | Cdk8 | Cyclin Dependent Kinase 8; member of the cyclin-dependent protein kinase (CDK) family. CDK family members are known to be important regulators of cell cycle progression. This kinase and its regulatory subunit, cyclin C, are components of the Mediator transcriptional regulatory complex, involved in both transcriptional activation and repression by phosphorylation of the carboxy-terminal domain of the largest subunit of RNA polymerase II. This kinase regulates transcription by targeting the cyclin-dependent kinase 7 subunits of the general transcription initiation factor IIH, thus providing a link between the Mediator complex and the basal transcription machinery. Multiple pseudogenes of this gene have been identified. Alternative splicing results in multiple transcript variants. | 1,9 |
|  | 6 | Cyclin A1 (C-term) | Cyclin A1; encoded by this gene belongs to the highly conserved cyclin family, whose members are characterized by a dramatic periodicity in protein abundance through the cell cycle. Cyclins function as regulators of CDK kinases. Different cyclins exhibit distinct expression and degradation patterns which contribute to the temporal coordination of each mitotic event. The cyclin encoded by this gene was shown to be expressed in testis and brain, as well as in several leukemic cell lines, and is thought to primarily function in the control of the germline meiotic cell cycle. This cyclin binds both CDK2 and CDC2 kinases, which give two distinct kinase activities, one appearing in S phase, the other in G2, and thus regulate separate functions in cell cycle. This cyclin was found to bind to important cell cycle regulators, such as Rb family proteins, transcription factor E2F-1, and the p21 family proteins. | 1,8 |
|  | 7 | p19ARF | Cyclin Dependent Kinase Inhibitor 2A, CDKN2A; This ARF product functions as a stabilizer of the tumor suppressor protein p53 as it can interact with, and sequester, the E3 ubiquitin-protein ligase MDM2, a protein responsible for the degradation of p53. In | 1,8 |
|  | 8 | NuMA | Nuclear Mitotic Apparatus Protein 1; a large protein that forms a structural component of the nuclear matrix. The encoded protein interacts with microtubules and plays a role in the formation and organization of the mitotic spindle during cell division. | 1,7 |
|  | 9 | Glycogen Synthase Kinase 3b (GSK3b) | Glycogen Synthase Kinase 3 Beta; It is a negative regulator of glucose homeostasis and is involved in energy metabolism, inflammation, ER-stress, mitochondrial dysfunction, and apoptotic pathways. | 1,6 |
|  | 10 | Cyclin B1 | Cyclin B1; protein encoded by this gene is a regulatory protein involved in mitosis. The gene product complexes with p34(cdc2) to form the maturation-promoting factor (MPF). The encoded protein is necessary for proper control of the G2/M transition phase of the cell cycle. | 1,6 |
|  | 11 | p73 | Tumor Protein P73; encodes a member of the p53 family of transcription factors involved in cellular responses to stress and development. It maps to a region on chromosome 1p36 that is frequently deleted in neuroblastoma and other tumors, and thought to contain multiple tumor suppressor genes. The demonstration that this gene is monoallelically expressed (likely from the maternal allele), supports the notion that it is a candidate gene for neuroblastoma. Many transcript variants resulting from alternative splicing and/or use of alternate promoters have been found for this gene, but the biological validity and the full-length nature of some variants have not been determined. | 1,6 |
|  | 12 | 14.3.3, Pan | 14-3-3 proteins are a family of small, widely expressed, highly conserved cytosolic proteins. 14-3-3 proteins bind to and influence the activities of a diverse group of molecules involved in signal transduction, cell cycle regulation and apoptosis, including Raf, PKC, Bad, Cbl, and c-Bcr. Interactions between 14-3-3 and target proteins are strongly influenced by the phosphorylation state of 14-3-3 and the target protein. | 1,6 |
|  | 13 | p19Skp1 | S-Phase Kinase Associated Protein 1, SKP1; encodes a component of SCF complexes, which are composed of this protein, cullin 1, a ring-box protein, and one member of the F-box family of proteins. This protein binds directly to the F-box motif found in F-box proteins. SCF complexes are involved in the regulated ubiquitination of specific protein substrates, which targets them for degradation by the proteosome. Specific F-box proteins recognize different target protein(s), and many specific SCF substrates have been identified including regulators of cell cycle progression and development. Studies have also characterized the protein as an RNA polymerase II elongation factor. Alternative splicing of this gene results in two transcript variants. | 1,5 |
|  | 14 | p35nck5a | Cyclin Dependent Kinase 5 Regulatory Subunit 1; encoded by this gene (p35) is a neuron-specific activator of cyclin-dependent kinase 5 (CDK5); the activation of CDK5 is required for proper development of the central nervous system. The p35 form of this protein is proteolytically cleaved by calpain, generating a p25 form. The cleavage of p35 into p25 results in relocalization of the protein from the cell periphery to nuclear and perinuclear regions. P25 deregulates CDK5 activity by prolonging its activation and changing its cellular location. The p25 form accumulates in the brain neurons of patients with Alzheimer's disease. This accumulation correlates with an increase in CDK5 kinase activity, and may lead to aberrantly phosphorylated forms of the microtubule-associated protein tau, which contributes to Alzheimer's disease. | 1,4 |
|  | 15 | c-Abl | ABL Proto-Oncogene 1; Abelson Tyrosine-Protein Kinase 1; protein tyrosine kinase involved in a variety of cellular processes, including cell division, adhesion, differentiation, and response to stress. | 1,4 |
|  | 16 | Beta actin | ACTB; Actins are highly conserved proteins that are involved in cell motility, structure, integrity, and intercellular signaling; n addition to their role in the cytoplasmic cytoskeleton, G- and F-actin also localize in the nucleus, and regulate gene transcription and motility and repair of damaged DNA | 1,4 |
|  | 17 | Cdk7 | Cyclin Dependent Kinase 7; member of the cyclin-dependent protein kinase (CDK) family. CDK family members are highly similar to the gene products of Saccharomyces cerevisiae cdc28, and Schizosaccharomyces pombe cdc2, and are known to be important regulators of cell cycle progression. This protein forms a trimeric complex with cyclin H and MAT1, which functions as a Cdk-activating kinase (CAK). It is an essential component of the transcription factor TFIIH, that is involved in transcription initiation and DNA repair. This protein is thought to serve as a direct link between the regulation of transcription and the cell cycle. | 1,4 |
|  | 18 | Cyclin A(A1/A2) (inter) | Cyclin A1; encoded by this gene belongs to the highly conserved cyclin family, whose members are characterized by a dramatic periodicity in protein abundance through the cell cycle. Cyclins function as regulators of CDK kinases. Different cyclins exhibit distinct expression and degradation patterns which contribute to the temporal coordination of each mitotic event. The cyclin encoded by this gene was shown to be expressed in testis and brain, as well as in several leukemic cell lines, and is thought to primarily function in the control of the germline meiotic cell cycle. This cyclin binds both CDK2 and CDC2 kinases, which give two distinct kinase activities, one appearing in S phase, the other in G2, and thus regulate separate functions in cell cycle. This cyclin was found to bind to important cell cycle regulators, such as Rb family proteins, transcription factor E2F-1, and the p21 family proteins. | 1,3 |
|  | 19 | p130 (RBL2) | RB Transcriptional Corepressor Like 2; Key regulator of entry into cell division. Directly involved in heterochromatin formation by maintaining overall chromatin structure and, in particular, that of constitutive heterochromatin by stabilizing histone methylation. Recruits and targets histone methyltransferases KMT5B and KMT5C, leading to epigenetic transcriptional repression. | 1,3 |
|  | 20 | p73a | Tumor Protein P73; encodes a member of the p53 family of transcription factors involved in cellular responses to stress and development. It maps to a region on chromosome 1p36 that is frequently deleted in neuroblastoma and other tumors, and thought to contain multiple tumor suppressor genes. The demonstration that this gene is monoallelically expressed (likely from the maternal allele), supports the notion that it is a candidate gene for neuroblastoma. Many transcript variants resulting from alternative splicing and/or use of alternate promoters have been found for this gene, but the biological validity and the full-length nature of some variants have not been determined. | 1,3 |
|  | 21 | CDC34 | Cell Division Cycle 34, Ubiqiutin Conjugating Enzyme; This protein is a part of the large multiprotein complex, which is required for ubiquitin-mediated degradation of cell cycle G1 regulators, and for the initiation of DNA replication. | 1,3 |
|  | 22 | Cyclin C | Cyclin C; protein encoded by this gene is a member of the cyclin family of proteins. The encoded protein interacts with cyclin-dependent kinase 8 and induces the phophorylation of the carboxy-terminal domain of the large subunit of RNA polymerase II. The level of mRNAs for this gene peaks in the G1 phase of the cell cycle. Two transcript variants encoding different isoforms have been found for this gene. | 1,3 |
|  | 23 | CDC37 | Cell Division Cycle 37, HSP90 Cochaperone; highly similar to Cdc 37, a cell division cycle control protein of Sacchromyces cerevisiae. This protein is a molecular chaperone with specific function in cell signal transduction. It has been shown to form complex with Hsp90 and a variety of protein kinases including CDK4, CDK6, SRC, RAF-1, MOK, as well as eIF2 alpha kinases. It is thought to play a critical role in directing Hsp90 to its target kinases. Cdc37 is essential for chromosome segregation and cytokinesis in higher eukaryotes. | 1,3 |
|  | 24 | GAPDH | Glyceraldehyde-3-Phosphate Dehydrogenase; member of the glyceraldehyde-3-phosphate dehydrogenase protein family. The encoded protein has been identified as a moonlighting protein based on its ability to perform mechanistically distinct functions. The product of this gene catalyzes an important energy-yielding step in carbohydrate metabolism, the reversible oxidative phosphorylation of glyceraldehyde-3-phosphate in the presence of inorganic phosphate and nicotinamide adenine dinucleotide (NAD). The encoded protein has additionally been identified to have uracil DNA glycosylase activity in the nucleus. Also, this protein contains a peptide that has antimicrobial activity against E. coli, P. aeruginosa, and C. albicans. Studies of a similar protein in mouse have assigned a variety of additional functions including nitrosylation of nuclear proteins, the regulation of mRNA stability, and acting as a transferrin receptor on the cell surface of macrophage. | 1,2 |
|  | 25 | Tubulin-b | Tubulin Beta Class I; TUBB; This gene encodes a beta tubulin protein. This protein forms a dimer with alpha tubulin and acts as a structural component of microtubules. Mutations in this gene cause cortical dysplasia, complex, with other brain malformations 6. | 1,2 |
|  | 26 | Cdk1/p34cdc2 | Cyclin Dependent Kinase 1; protein encoded by this gene is a member of the Ser/Thr protein kinase family. This protein is a catalytic subunit of the highly conserved protein kinase complex known as M-phase promoting factor (MPF), which is essential for G1/S and G2/M phase transitions of eukaryotic cell cycle. Mitotic cyclins stably associate with this protein and function as regulatory subunits. The kinase activity of this protein is controlled by cyclin accumulation and destruction through the cell cycle. The phosphorylation and dephosphorylation of this protein also play important regulatory roles in cell cycle control. | 1,2 |
|  | 27 | Ki67 | Marker Of Proliferation Ki-67; Required to maintain individual mitotic chromosomes dispersed in the cytoplasm following nuclear envelope disassembly (PubMed:27362226). Associates with the surface of the mitotic chromosome, the perichromosomal layer, and covers a substantial fraction of the chromosome surface (PubMed:27362226). Prevents chromosomes from collapsing into a single chromatin mass by forming a steric and electrostatic charge barrier: the protein has a high net electrical charge and acts as a surfactant, dispersing chromosomes and enabling independent chromosome motility (PubMed:27362226). Binds DNA, with a preference for supercoiled DNA and AT-rich DNA (PubMed:10878551). | 1,2 |
|  | 28 | p18INK4c | Cyclin Dependent Kinase Inhibitor 2C, CDKN2C; member of the INK4 family of cyclin-dependent kinase inhibitors. This protein has been shown to interact with CDK4 or CDK6, and prevent the activation of the CDK kinases, thus function as a cell growth regulator that controls cell cycle G1 progression. Ectopic expression of this gene was shown to suppress the growth of human cells in a manner that appears to correlate with the presence of a wild-type RB1 function. Studies in the knockout mice suggested the roles of this gene in regulating spermatogenesis, as well as in suppressing tumorigenesis. | 1,2 |
|  | 29 | Cdk2 | Cyclin Dependent Kinase 2; protein is the catalytic subunit of the cyclin-dependent protein kinase complex, which regulates progression through the cell cycle. Activity of this protein is especially critical during the G1 to S phase transition. This protein associates with and regulated by other subunits of the complex including cyclin A or E, CDK inhibitor p21Cip1 (CDKN1A), and p27Kip1 (CDKN1B). | 1,2 |
|  | 30 | Cullin-2 (CUL-2) | Cullin 2; Core component of multiple cullin-RING-based ECS (ElonginB/C-CUL2/5-SOCS-box protein) E3 ubiquitin-protein ligase complexes, which mediate the ubiquitination of target proteins. ECS complexes and ARIH1 collaborate in tandem to mediate ubiquitination of target proteins | 1,2 |
|  | 31 | p16INK4a | Cyclin Dependent Kinase Inhibitor 2A, CDKN2A; This ARF product functions as a stabilizer of the tumor suppressor protein p53 as it can interact with, and sequester, the E3 ubiquitin-protein ligase MDM2, a protein responsible for the degradation of p53. In | 1,2 |
|  | 32 | CDC47 | MCM7; Minichromosome Maintenance Complex Component 7; that are essential for the initiation of eukaryotic genome replication. The hexameric protein complex formed by the MCM proteins is a key component of the pre-replication complex (pre_RC) and may be involved in the formation of replication forks and in the recruitment of other DNA replication related proteins. The MCM complex consisting of this protein and MCM2, 4 and 6 proteins possesses DNA helicase activity, and may act as a DNA unwinding enzyme. Cyclin D1-dependent kinase, CDK4, is found to associate with this protein, and may regulate the binding of this protein with the tumorsuppressor protein RB1/RB. | 1,2 |
|  | 33 | PCNA | Proliferating Cell Nuclear Antigen; encoded by this gene is found in the nucleus and is a cofactor of DNA polymerase delta. The encoded protein acts as a homotrimer and helps increase the processivity of leading strand synthesis during DNA replication. In response to DNA damage, this protein is ubiquitinated and is involved in the RAD6-dependent DNA repair pathway. | 1,2 |

Supplementary table S3: Downregulation of cell cycle target genes by HPL-based culture conditions (≤ 1.2 fold) for each tissue source ranked from highest to lowest (FC = fold change, for all data shown p < 0.05).

| Stromal cell type | *#* | *Protein name* | *Function according to genecards data base entry* | *FC* |
| --- | --- | --- | --- | --- |
| **BM** | 1 | Mitochondria Ab-2 | Ab-2 recognizes a 60kDa nonglycosylated protein component of mitochondria found in human cells. It can be used to stain the mitochondria in cell or tissue preparations and can be used as a marker of the mitochondria in subcellular fractions. This monoclonal antibody is part of a new panel of reagents, which recognizes subcellular organelles or compartments of human cells. These markers may be useful in identification of these organelles in cells, tissues, and biochemical preparations. MAb-2 produces a "spaghetti"-like staining pattern in the cytoplasm of normal and malignant human cells and may be used to stain the mitochondria in paraformaldehyde fixed frozen and formalin-fixed, paraffin-embedded tissues. | -1,8 |
| **UC** | 1 | Retinoblastoma | RB Transcriptional Corepressor 1; Retinoblastoma 1; encoded by this gene is a negative regulator of the cell cycle and was the first tumor suppressor gene found. The encoded protein also stabilizes constitutive heterochromatin to maintain the overall chromatin structure. The active, hypophosphorylated form of the protein binds transcription factor E2F1; Key regulator of entry into cell division that acts as a tumor suppressor. Promotes G0-G1 transition when phosphorylated by CDK3/cyclin-C. Acts as a transcription repressor of E2F1 target genes. | -1,2 |
|  | 2 | Cyclin E | Cyclin E1; belongs to the highly conserved cyclin family, whose members are characterized by a dramatic periodicity in protein abundance through the cell cycle. Cyclins function as regulators of CDK kinases. Different cyclins exhibit distinct expression and degradation patterns which contribute to the temporal coordination of each mitotic event. This cyclin forms a complex with and functions as a regulatory subunit of CDK2, whose activity is required for cell cycle G1/S transition. This protein accumulates at the G1-S phase boundary and is degraded as cells progress through S phase. Overexpression of this gene has been observed in many tumors, which results in chromosome instability, and thus may contribute to tumorigenesis. This protein was found to associate with, and be involved in, the phosphorylation of NPAT protein (nuclear protein mapped to the ATM locus), which participates in cell-cycle regulated histone gene expression and plays a critical role in promoting cell-cycle progression in the absence of pRB. | -1,2 |
|  | 3 | PCNA | Proliferating Cell Nuclear Antigen; encoded by this gene is found in the nucleus and is a cofactor of DNA polymerase delta. The encoded protein acts as a homotrimer and helps increase the processivity of leading strand synthesis during DNA replication. In response to DNA damage, this protein is ubiquitinated and is involved in the RAD6-dependent DNA repair pathway. | -1,2 |
|  | 4 | Tubulin-b | Tubulin Beta Class I; TUBB; This gene encodes a beta tubulin protein. This protein forms a dimer with alpha tubulin and acts as a structural component of microtubules. Mutations in this gene cause cortical dysplasia, complex, with other brain malformations 6. | -1,3 |
|  | 5 | APC2 | ANAPC2; Anaphase Promoting Complex Subunit 2; A large protein complex, termed the anaphase-promoting complex (APC), or the cyclosome, promotes metaphase-anaphase transition by ubiquitinating its specific substrates such as mitotic cyclins and anaphase inhibitor, which are subsequently degraded by the 26S proteasome. Biochemical studies have shown that the vertebrate APC contains eight subunits. | -1,3 |
|  | 6 | p53 | Tumor Protein P53; encodes a tumor suppressor protein containing transcriptional activation, DNA binding, and oligomerization domains. The encoded protein responds to diverse cellular stresses to regulate expression of target genes, thereby inducing cell cycle arrest, apoptosis, senescence, DNA repair, or changes in metabolism. Mutations in this gene are associated with a variety of human cancers, including hereditary cancers such as Li-Fraumeni syndrome. Alternative splicing of this gene and the use of alternate promoters result in multiple transcript variants and isoforms. | -1,3 |
|  | 7 | Mitochondria Ab-2 | Ab-2 recognizes a 60kDa nonglycosylated protein component of mitochondria found in human cells. It can be used to stain the mitochondria in cell or tissue preparations and can be used as a marker of the mitochondria in subcellular fractions. This monoclonal antibody is part of a new panel of reagents, which recognizes subcellular organelles or compartments of human cells. These markers may be useful in identification of these organelles in cells, tissues, and biochemical preparations. MAb-2 produces a "spaghetti"-like staining pattern in the cytoplasm of normal and malignant human cells and may be used to stain the mitochondria in paraformaldehyde fixed frozen and formalin-fixed, paraffin-embedded tissues. | -2,1 |
| **WAT** | 1 | ATM | ATM Serine/Threonine Kinase; is an important cell cycle checkpoint kinase that phosphorylates; thus, it functions as a regulator of a wide variety of downstream proteins, including tumor suppressor proteins p53 and BRCA1, checkpoint kinase CHK2, checkpoint proteins RAD17 and RAD9, and DNA repair protein NBS1. This protein and the closely related kinase ATR are thought to be master controllers of cell cycle checkpoint signaling pathways that are required for cell response to DNA damage and for genome stability. | -1,2 |
|  | 2 | p130cas | BCAR1 Scaffold Protein, Cas Family Member; member of the Crk-associated substrate (CAS) family of scaffold proteins, characterized by the presence of multiple protein-protein interaction domains and many serine and tyrosine phosphorylation sites. The encoded protein contains a Src-homology 3 (SH3) domain, a proline-rich domain, a substrate domain which contains 15 repeat of the YxxP consensus phosphorylation motif for Src family kinases, a serine-rich domain, and a bipartite Src-binding domain, which can bind both SH2 and SH3 domains. This adaptor protein functions in multiple cellular pathways, including in cell motility, apoptosis and cell cycle control. | -1,2 |
|  | 3 | E2F-1 | E2F Transcription Factor 1; member of the E2F family of transcription factors. The E2F family plays a crucial role in the control of cell cycle and action of tumor suppressor proteins and is also a target of the transforming proteins of small DNA tumor viruses. The E2F proteins contain several evolutionally conserved domains found in most members of the family. These domains include a DNA binding domain, a dimerization domain which determines interaction with the differentiation regulated transcription factor proteins (DP), a transactivation domain enriched in acidic amino acids, and a tumor suppressor protein association domain which is embedded within the transactivation domain. This protein and another 2 members, E2F2 and E2F3, have an additional cyclin binding domain. This protein binds preferentially to retinoblastoma protein pRB in a cell-cycle dependent manner. It can mediate both cell proliferation and p53-dependent/independent apoptosis. | -1,3 |
|  | 4 | RAD 51 | RAD51 Recombinase; encoded by this gene is a member of the RAD51 protein family. RAD51 family members are highly similar to bacterial RecA and Saccharomyces cerevisiae Rad51, and are known to be involved in the homologous recombination and repair of DNA. This protein can interact with the ssDNA-binding protein RPA and RAD52, and it is thought to play roles in homologous pairing and strand transfer of DNA. This protein is also found to interact with BRCA1 and BRCA2, which may be important for the cellular response to DNA damage. BRCA2 is shown to regulate both the intracellular localization and DNA-binding ability of this protein. Loss of these controls following BRCA2 inactivation may be a key event leading to genomic instability and tumorigenesis. | -1,4 |
|  | 5 | Chk1 | CHEK1; Checkpoint Kinase 1; gene belongs to the Ser/Thr protein kinase family. It is required for checkpoint mediated cell cycle arrest in response to DNA damage or the presence of unreplicated DNA. This protein acts to integrate signals from ATM and ATR, two cell cycle proteins involved in DNA damage responses, that also associate with chromatin in meiotic prophase I. Phosphorylation of CDC25A protein phosphatase by this protein is required for cells to delay cell cycle progression in response to double-strand DNA breaks. | -1,8 |
|  | 6 | Mitochondria Ab-2 | Ab-2 recognizes a 60kDa nonglycosylated protein component of mitochondria found in human cells. It can be used to stain the mitochondria in cell or tissue preparations and can be used as a marker of the mitochondria in subcellular fractions. This monoclonal antibody is part of a new panel of reagents, which recognizes subcellular organelles or compartments of human cells. These markers may be useful in identification of these organelles in cells, tissues, and biochemical preparations. MAb-2 produces a "spaghetti"-like staining pattern in the cytoplasm of normal and malignant human cells and may be used to stain the mitochondria in paraformaldehyde fixed frozen and formalin-fixed, paraffin-embedded tissues. | -5,9 |
