# Supplementary material for: Transcription Factors STAT3 and MYC Are Key Players of Human Platelet Lysate-Induced Cell Proliferation
Source: Int J Mol Sci. 2022 Dec 13;23(24):15782. doi: 10.3390/ijms232415782 (PMC9781157; doi:10.3390/ijms232415782)
Supplement: Supplementary file 1 [file ijms-23-15782-s001.zip › ijms-2054186-supplementary/Supplementary_material/Supplementary_table_S4.docx]

Supplementary table S4: Primer sequences used for quantitative RT-qPCR.

| Gene | Primer | sequence (5‘-3‘) |
| --- | --- | --- |
| MYC  MYC Proto-Oncogene, BHLH Transcription Factor | forward | GGACGACGAGACCTTCATCAA |
|  | reverse | CCAGCTTCTCTGAGACGAGCTT |
| STAT3  Signal transducer and activator of transcription 3 | forward | GGTACATCATGGGCTTTATC |
|  | reverse | TTTGCTGCTTTCACTGAATC |
| ABL1  ABL Proto-Oncogene 1 | forward | GCATTTGGAGTATTGCTTTG |
|  | reverse | CATGAGTTCATAGACCTTCTC |
| APC2  Adenomatous Polyposis Coli Protein 2 | forward | ACCTACAGGGAAAACTGG |
|  | reverse | GTGATGTCCATCTGTAGGG |
| CCNA1  Cyclin A1 | forward | TTATCAGTACCTTAGGGAAGC |
|  | reverse | CAGACATACATGAAAGGAACC |
| CCNB1  Cyclin B1 | forward | AGAGCATCTAAGATTGGAGAG |
|  | reverse | CCATGTCATAGTCCAACATAG |
| CCND1  Cyclin D1 | forward | GCCTCTAAGATGAAGGAGAC |
|  | reverse | CCATTTGCAGCAGCTC |
| CCNE1  Cyclin E1 | forward | AGACATACTTAAGGGATCAGC |
|  | reverse | CACACCTCCATTAACCAATC |
| CDK2  Cyclin-dependent kinase 2 | forward | GAGACCTTAAACCTCAGAATC |
|  | reverse | TGGAATAATATTTGCAGCCC |
| CDK3  Cyclin-dependent kinase 3 | forward | CATGGATATGTTCCAGAAGG |
|  | reverse | CCAAATCCAGTCTGATCTTC |
| CDKN1B – p27  cyclin-dependent kinase inhibitor 1B | forward | AAAATGTTTCAGACGGTTCC |
|  | reverse | ATTCGAGCTGTTTACGTTTG |
| NUMA  Nuclear Mitotic Apparatus Protein 1 | forward | AATCGAAAACATCCCTCTTC |
|  | reverse | TAGAGTGGTATAAGAGCAGC |
