# Supplementary material for: Transcription Factors STAT3 and MYC Are Key Players of Human Platelet Lysate-Induced Cell Proliferation
Source: Int J Mol Sci. 2022 Dec 13;23(24):15782. doi: 10.3390/ijms232415782 (PMC9781157; doi:10.3390/ijms232415782)
Supplement: Supplementary file 1 [file ijms-23-15782-s001.zip › ijms-2054186-supplementary/Supplementary_material/Supplementary_table_S5.docx]

Supplementary table S5: List of cell cycle specific antibodies used for ELISA-based antibody microarray.

| **ID** | **Antibody Name** | **IG Isotype** | **Epitope** | **Source** |
| --- | --- | --- | --- | --- |
| 1 | 14.3.3, Pan | IgG1 / κ | Not determined | Mouse |
| 2 | APC11 | N/A | aa 76-84 | Rabbit |
| 3 | APC2 | N/A | aa 76-84 | Rabbit |
| 4 | ATM | IgG1 | C-terminal | Mouse |
| 5 | Beta actin | N/A | Not determined | Mouse |
| 6 | c-Abl | IgG1 / γ | SH2 domain | Mouse |
| 7 | CDC14A Phosphatase | IgG1 | C-terminal | Mouse |
| 8 | CDC25C | IgG1 / κ | aa 1-150 | Mouse |
| 9 | CDC34 | N/A | aa285-298 | Rabbit |
| 10 | CDC37 | N/A | aa 369-379 | Rabbit |
| 11 | CDC47 | IgG1 / κ | Not determined | Rabbit |
| 12 | CDC6 | IgG1 | Not determined | Mouse |
| 13 | cdh1 | IgG1 / κ | Not determined | Mouse |
| 14 | Cdk1/p34^cdc2^ | IgG2a | aa220-227 | Mouse |
| 15 | Cdk2 | IgG2b | Not determined | Mouse |
| 16 | Cdk3 | N/A | aa 290-305 | Rabbit |
| 17 | Cdk4 | IgG 1 / κ | aa 1-20 | Mouse |
| 18 | Cdk5 | IgG1 | Not determined | Mouse |
| 19 | Cdk7 | IgG2b | 221 aa fragment of C-terminus | Mouse |
| 20 | Cdk8 | N/A | aa 451-464 | Rabbit |
| 21 | Chk1 | IgG2b / κ | Not determined | Mouse |
| 22 | Cullin-1 (CUL-1) | N/A | aa 742-752 | Rabbit |
| 23 | Cullin-2 (CUL-2) | N/A | aa 733-745 | Rabbit |
| 24 | Cullin-3 (CUL-3) | N/A | N-terminal | Rabbit |
| 25 | Cyclin A(A1/A2) (inter) | N/A | Not determined | Mouse |
| 26 | Cyclin A1 (C-term) | N/A | Not determined | Mouse |
| 27 | Cyclin B1 | N/A | C-terminal | Rabbit |
| 28 | Cyclin C | N/A | aa 290-303 | Rabbit |
| 29 | Cyclin D1 | N/A | C-terminus | Rabbit |
| 30 | Cyclin D3 | IgG1 / κ | Not determined | Mouse |
| 31 | Cyclin E | N/A | C-terminal | Rabbit |
| 32 | Cyclin E2 | N/A | aa 391-404 | Rabbit |
| 33 | E2F-1 | IgG2a / κ | aa1-89 | Mouse |
| 34 | E2F-2 | IgG1 | aa 1-85 | Mouse |
| 35 | E2F-3 | IgG2b | aa 1-132 | Mouse |
| 36 | GAPDH | N/A | Not determined | Mouse |
| 37 | Glycogen Synthase Kinase 3b (GSK3b) | N/A | Kinase subdomain XI region | Rabbit |
| 38 | Ki67 | N/A | Middle of Ki67 protein | Rabbit |
| 39 | Mitochondria | IgG1 | Not determined | Mouse |
| 40 | NuMA | IgM / κ | Not determined | Mouse |
| 41 | p130 | IgG1 | aa 878-913 | Mouse |
| 42 | p130^cas^ | IgG1 / κ | Not determined | Mouse |
| 43 | p14^ARF^ | N/A | aa 119-132 | Rabbit |
| 44 | p15^INK4b^ | IgG1 / κ | Not determined | Mouse |
| 45 | p16^INK4a^ | IgG1 | Not determined | Mouse |
| 46 | p18^INK4c^ | N/A | aa 155-168 | Rabbit |
| 47 | p19^ARF^ | N/A | Middle of p19ARF | Rabbit |
| 48 | p19^Skp1^ | N/A | aa 152-163 | Rabbit |
| 49 | p21^WAF1^ | IgG2a | Not determined | Mouse |
| 50 | p27^Kip1^ | IgG1 / κ | aa 83-197 | Mouse |
| 51 | p35^nck5a^ | IgG2b / κ | Not determined | Mouse |
| 52 | p53 | IgG1 | aa 212-217 | Mouse |
| 53 | p57^Kip2^ | N/A | Near C-terminus | Rabbit |
| 54 | p73 | N/A | aa1-15 | Rabbit |
| 55 | p73a | IgG1 | aa380-637 (p73α) | Mouse |
| 56 | p73a/b | IgG1 / κ | aa380-637 (p73α) | Mouse |
| 57 | PCNA | IgG2a / κ | Not determined | Mouse |
| 58 | RAD 51 | IgG1 / κ | Not determined | Mouse |
| 59 | Retinoblastoma | IgG1 | aa703-722 | Mouse |
| 60 | Retinoblastoma (Rb) (Phospho-specific Serine^608^) | IgG1 | Not determined | Mouse |
| 61 | ROC | N/A | aa 97-108 | Rabbit |
| 62 | Topo II beta | N/A | 300 aa fragment of C-terminus | Rabbit |
| 63 | Tubulin-a | IgG1 / κ | aa 426-450 | Mouse |
| 64 | Tubulin-b | IgM | Not determined | Mouse |
